# *Lactobacillus rossiae*, a Vitamin B_12_ Producer, Represents a Metabolically Versatile Species within the Genus *Lactobacillus*

**DOI:** 10.1371/journal.pone.0107232

**Published:** 2014-09-29

**Authors:** Maria De Angelis, Francesca Bottacini, Bruno Fosso, Philip Kelleher, Maria Calasso, Raffaella Di Cagno, Marco Ventura, Ernesto Picardi, Douwe van Sinderen, Marco Gobbetti

**Affiliations:** 1 Department of Soil, Plant and Food Sciences, University of Bari Aldo Moro, Bari, Italy; 2 Department of Microbiology, University College Cork, Cork, Ireland; 3 Department of Bioscience, Biotechnology and Biopharmaceutical, University of Bari Aldo Moro, Bari, Italy; 4 Laboratory of Probiogenomics, Department of Life Sciences, University of Parma, Parma, Italy; 5 Institute of Biomembranes and Bioenergetics (IBBE), CNR, Bari, Italy; 6 National Institute of Biostructures and Biosystems (INBB), Rome, Italy; 7 Alimentary Pharmabiotic Centre, University College Cork, Cork, Ireland; University of Florida, United States of America

## Abstract

*Lactobacillus rossiae* is an obligately hetero-fermentative lactic acid bacterium, which can be isolated from a broad range of environments including sourdoughs, vegetables, fermented meat and flour, as well as the gastrointestinal tract of both humans and animals. In order to unravel distinctive genomic features of this particular species and investigate the phylogenetic positioning within the genus *Lactobacillus*, comparative genomics and phylogenomic approaches, followed by functional analyses were performed on *L. rossiae* DSM 15814^T^, showing how this type strain not only occupies an independent phylogenetic branch, but also possesses genomic features underscoring its biotechnological potential. This strain in fact represents one of a small number of bacteria known to encode a complete *de novo* biosynthetic pathway of vitamin B_12_ (in addition to other B vitamins such as folate and riboflavin). In addition, it possesses the capacity to utilize an extensive set of carbon sources, a characteristic that may contribute to environmental adaptation, perhaps enabling the strain's ability to populate different niches.

## Introduction

*Lactobacillus rossiae* (formerly *Lactobacillus rossii*, designated a novel species in 2005 [1],) is an obligately hetero-fermentative lactic acid bacterium, exhibiting close phylogenetic relationship with *Lactobacillus durianis*, *Lactobacillus malefermentans* and *Lactobacillus suebicus*. *L. rossiae* strains have been isolated from the microbiota of sourdoughs [1], [2], [3], [4], wheat-legume sourdough [5], spelt flour [6], pineapples [7] and fermented meat [8], and from the gastrointestinal tract of humans [9] and animals [10]. The ability of *Lactobacillus* spp. to adapt to different environments is variable: some species are isolated from diverse habitats, while others appear to be restricted to specific niches [11]. The latter situation applies to *Lactobacillus sanfranciscensis*, which has so far only been found in sourdoughs. In contrast, *Lactobacillus casei* and *Lactobacillus plantarum* represent more versatile species, as they have been isolated from various fermented foods and are natural inhabitants of the human gastrointestinal tract [11].

Comparative genome analysis of *Lactobacillus* species has indicated that environmental adaptations cause a combination of gene gain and gene loss events [12], [13], [14]. Adaptation to the dairy niche appears to be associated with a tendency towards metabolic simplification. Loss of genes involved in carbohydrate metabolism and biosynthesis of amino acids and cofactors, and acquisition of genes required for peptide transport and hydrolysis have indeed been described in lactic acid bacteria [14], [15]. Species such as *Lactobacillus gasseri* and *Lactobacillus johnsonii*, which are commonly found in the gastrointestinal tract, carry genetic features that contribute to gut survival and promote interactions with intestinal mucosa. *L. casei* and *L. plantarum* possess the largest chromosome (2.9 and 3.3 Mb, respectively) among lactic acid bacteria, and contain a large number of regulatory and transport functions. Similar to *L. plantarum*, strains of *L. rossiae* exhibit remarkable ecological adaptability, genotypic and phenotypic diversity, and may be used to ferment a variety of foods [3], [16]. Some strains have been identified based on their antifungal activity [17], [18], and/or used for sourdough biotechnology, glutamate production [19] and fermentation of wheat germ [20].

The peculiarities of food fermentations mainly depend on the physiological and biochemical traits of the processing microbiota. Substantial progress has been achieved in genomic sequencing of lactic acid bacteria, especially for those species that are used for making dairy, sourdough, meat and vegetable foods, or those with promising probiotic traits [11]. To date (June, 2014), genomes of 78 lactobacilli have been completely sequenced or their genome sequencing is in progress (source http://www.ncbi.nlm.nih.gov/genome). Knowledge on the genomic features of lactobacilli will facilitate the identification of species and/or strain-specific traits, which may allow the rational design of strategies for improved fermentations or probiotic formulations for targeted food matrices or human applications [21].

For the above reasons, this study carried out the genomic annotation, comparative analyses, and metabolic pathway reconstruction of *L. rossiae* DSM 15814^T^, in addition to phenotypic analyses to confirm some of the predicted functional features.

## Results and Discussion

### General genome features

**Figure 1:**
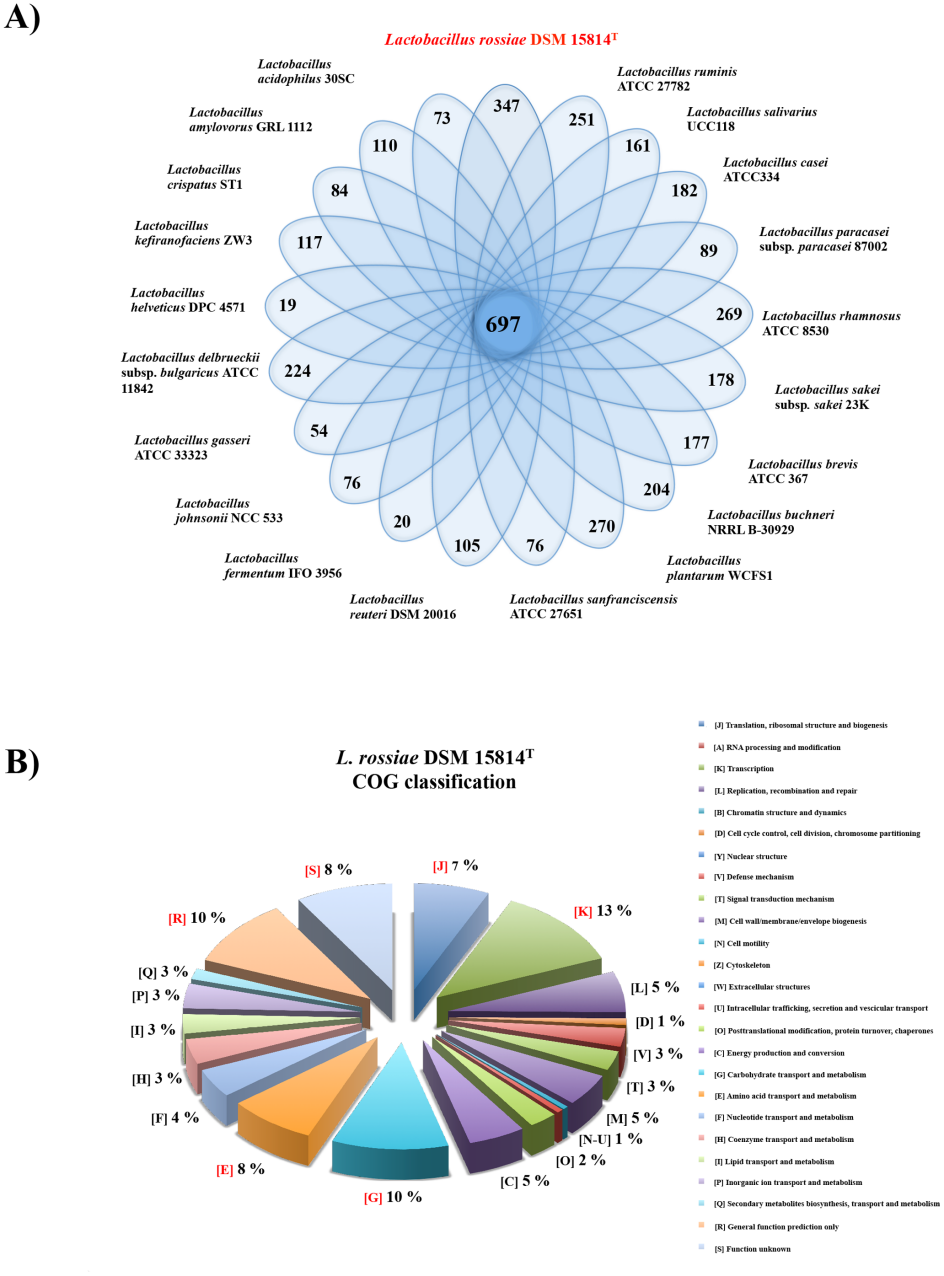
Classification of *Lactobacillus rossiae* DSM 15814^T^. A) Venn diagram displaying core gene families obtained by MCL clustering, and unique genes of *L. rossiae* DSM 15814^T^ and selected representatives of the *Lactobacillus* genus. B) Cluster of Orthologous Groups (COGs) classification of *L. rossiae* DSM 15814^T^ based on the predicted ORFs. The occurrence of each COG family relative to the total of classified genes is indicated as a frequency percentage.

Whole-genome shotgun sequencing results of *L. rossiae* DSM 15814^T^ [22] were retrieved from the NCBI public database; subsequent analyses focused on a selected subset of 57 contigs (size>400 bp) (Table S1). The combined DNA sequence of these 57 contigs represented 2,870,031 base pairs, which was thus presumed to be the minimum predicted genome size of this organism. The average G+C mol % content of the combined DNA sequences was determined to be 43%, consistent with the G+C mol % range of the *Lactobacillus* genus [21]. The sequences were subjected to ORF prediction, which resulted in the identification of 2,701 protein-encoding sequences (Table S2, general features). *In silico* annotation assigned a function to 74% of these deduced proteins, while the remaining 26% were deemed to represent hypothetical proteins. A Venn diagram, displaying core gene families, and the unique genes of *L. rossiae* DSM 15814^T^ and other representatives of the *Lactobacillus* genus, is shown in Figure 1A. The Cluster of Orthologous Group (COG) classification assigned a COG category to 78% of the identified ORFs. This analysis established that the largest proportions of predicted genes are involved in carbohydrate and amino acid transport and metabolism (10% and 8%, respectively), and in general or unknown functions (10% and 8%, respectively) (Figure 1B). Regarding mobile elements, *L. rossiae* DSM 15814^T^ appears to harbour a single plasmid (with a size of 57,494 bp), which contains genes predicted to encode a type I R/M system (LROS_2631-33), and proteins involved in conjugation (TraG/TraD/TraE), where the latter genes clustered together with genes specifying putative surface proteins (LROS_2616-25) and several hypothetical proteins (Figure S1). In addition, an apparently incomplete prophage (LROS_2487-2558), was identified that was integrated in the bacterial chromosome in the proximity of a predicted tRNA^Gln^ gene (data not shown).

### Phylogeny and comparative analyses

**Figure 2:**
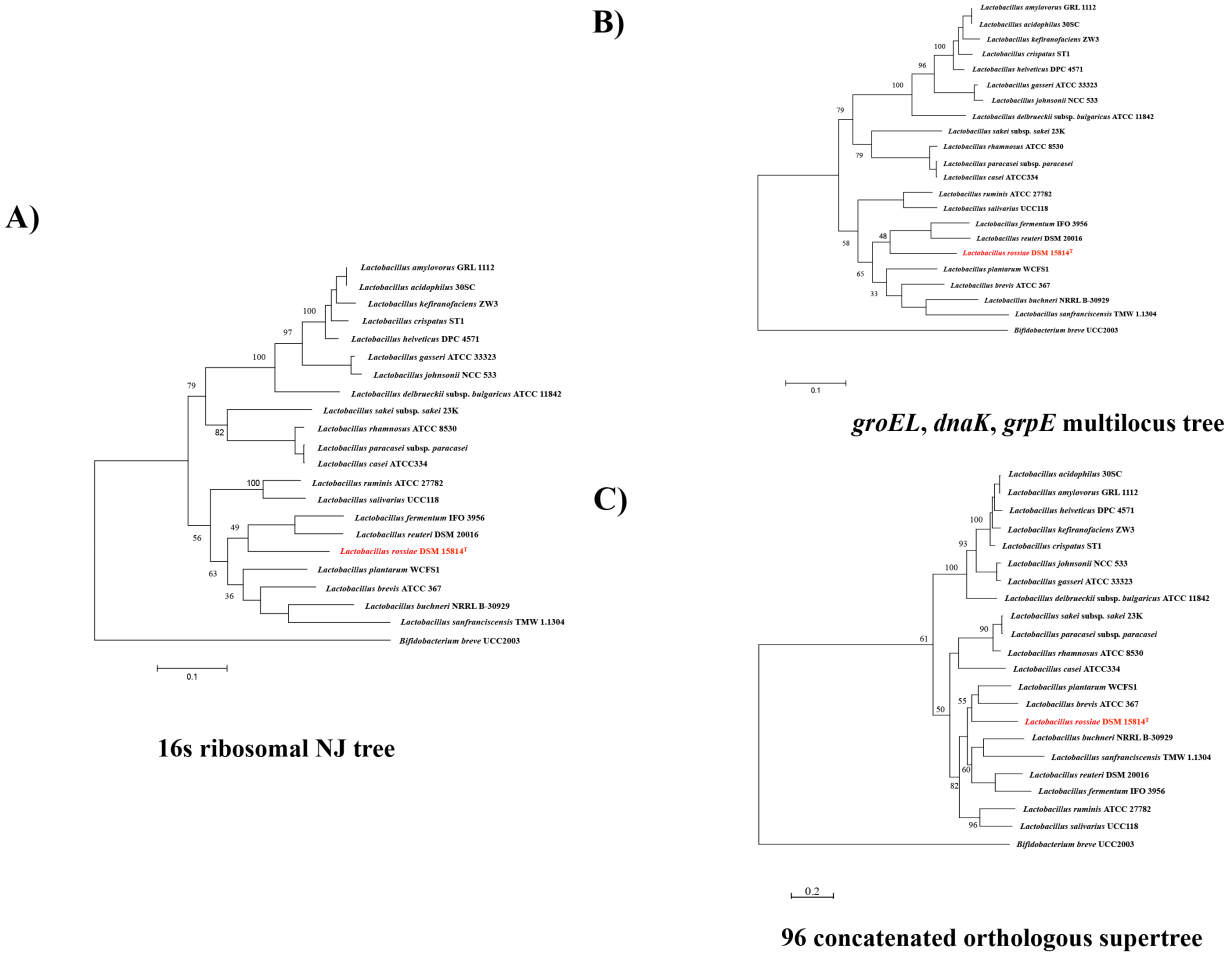
Phylogenomics of *Lactobacillus rossiae* DSM 15814^T^. A) 16S neighbor-joining (NJ) tree, resulting from the alignment of the 16S rRNA-encoding gene of *L. rossiae* DSM 15814^T^ and that of 20 selected representatives of the *Lactobacillus* genus (*L. amylovorus* GRL 1112, *L. acidophilus* 30SC, *L. kefiranofaciens* ZW3, *L. crispatus* ST1, *L. helveticus* DPC 4571, *L. gasseri* ATCC 33323, *L. johnsonii* NCC 533, *L. delbrueckii* subsp. *bulgaricus* ATCC 11842, *L. sakei* subsp. *sakei* 23K, *L. rhamnosus* ATCC 8530, *L. paracasei* subsp. *paracasei*, *L. casei* ATCC334, *L. ruminis* ATCC 27782, *L. salivarius* UCC118, *L. fermentum* IFO 3956, *L. reuteri* DSM 20016, *L. plantarum* WCFS1, *L. brevis* ATCC 367, *L. buchneri* NRRL B-30929 and *L. sanfranciscensis* TMW 1.1304). The corresponding 16S rRNA-specifying sequence of *Bifidobacterium breve* UCC2003 was used as an outgroup. The positioning of *L. rossiae* DSM 15814^T^ within the *Lactobacillus* genus is indicated in red. B) Multilocus supertree resulting from the alignment of the sequence of three housekeeping genes (*groEL*, *dnaK* and *grpE*) of *L. rossiae* DSM 15814^T^ and 20 selected representatives of the *Lactobacillus* genus (see above for the species). The positioning of *L. rossiae* DSM 15814^T^ within the *Lactobacillus* genus is indicated in red. C) Multilocus supertree resulting from the alignment of 96 selected orthologous genes of *L. rossiae* DSM 15814^T^ and 20 selected representatives of the *Lactobacillus* genus (see above for the species). The positioning of *L. rossiae* DSM 15814^T^ within the *Lactobacillus* genus is indicated in red.

**Figure 3:**
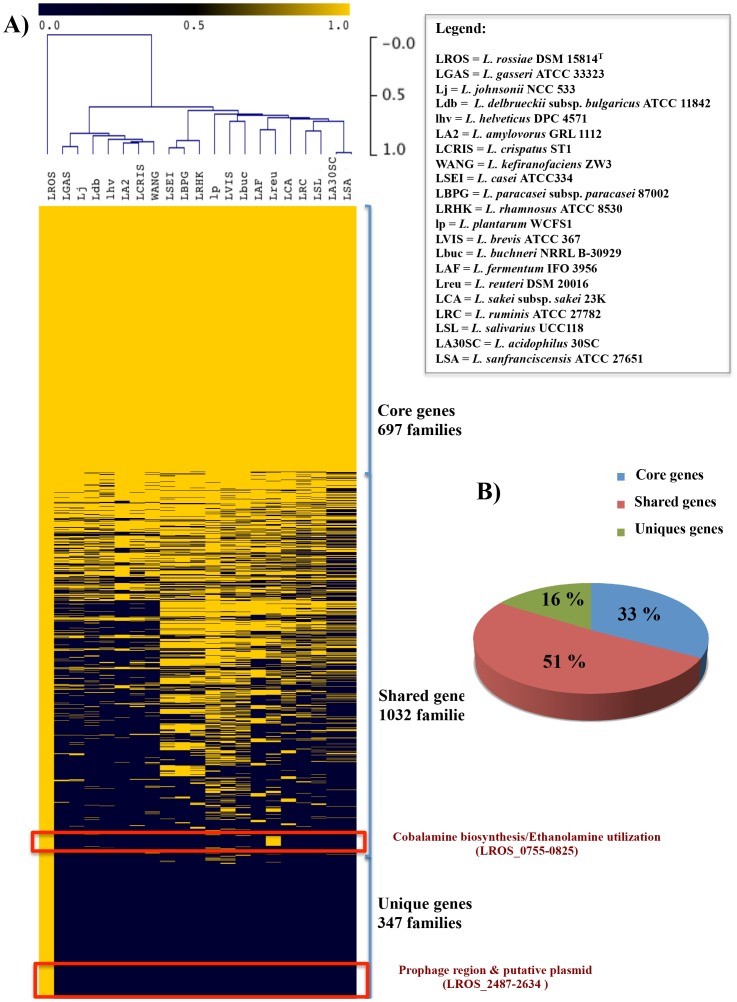
Comparative genomics of *L. rossiae* DSM 15814^T^. A) Hierarchical clustering analysis realized in Tmev and restricted to the sole variable genes of *L. rossiae* DSM 15814^T^ with respect to representatives of the *Lactobacillus* genus used for the analysis in Figure 2. The positioning of the cobalamin biosynthesis and ethanolamine utilization cluster, and of prophage region and putative plasmid are indicated. B) Pie chart indicating the core, extended core and variable genome with respect to the total of gene families, resulting from the MCL clustering algorithm.

To assess the phylogenetic positioning of *L. rossiae* within the *Lactobacillus* genus, a supertree based on the concatenation of 96 orthologous genes was built, using relevant sequences from the genomes of *L. rossiae* DSM 15814^T^, of 20 other fully sequenced *Lactobacillus* genus, and of *Bifidobacterium breve* UCC2003 as an outgroup (Figure 2A-C). This analysis revealed that the phylogenetic positioning of *L. rossiae* is close to that of the *L. plantarum* and *L. brevis* groups (Figure 2C), suggesting that these species may have followed similar strategies of environmental adaptation. BLASTP-based comparative analysis (Figure 3) showed that *L. rossiae* shares a high number of similar genes with *L. plantarum*, *L. brevis and L. buchneri,* thus confirming the higher degree of resolution using the 96 orthologous-based phylogenomic approach, compared to the one solely based on 16S rRNA gene sequence alignment (Figure 3A). The comparative genomics approach, coupled with hierarchical clustering analysis also allowed the definition of a set of 697 families (Figures 1A and 3A), which represent the predicted conserved core genome (gene families of *L. rossiae* DSM 15814^T^ which are also present in all other examined lactobacilli), accounting for 33% of the total of genes (Figure 3A-B). The remaining 67% of *L. rossiae* ORF content appeared instead to be variable (only present in the genomes of some, but not all examined lactobacilli), confirming that this species appears to be quite different from the other members of this genus. It furthermore allowed the identification of a gene cluster (designated here as the *pdu-cbi-cob-hem* cluster) putatively involved in cobalamin (vitamin B_12_) *de novo* biosynthesis, as well as the ethanolamine utilization cluster and the presence of various mobile elements (e.g., prophage region and a putative plasmid described above) (Figure 3).

### *De novo* production of cofactor vitamin B_12_ and related ethanolamine utilization

The comparative analysis conducted on the here identified *pdu-cbi-cob-hem* cluster of *L. rossiae* DSM 15814^T^ showed a high degree of homology with the previously characterized *pdu-cbi-cob-hem* cluster present in the genome of *L. reuteri* DSM 20026 [23], [24] and located upstream of a cobalamin-dependent ethanolamine utilization cluster.

**Figure 4:**
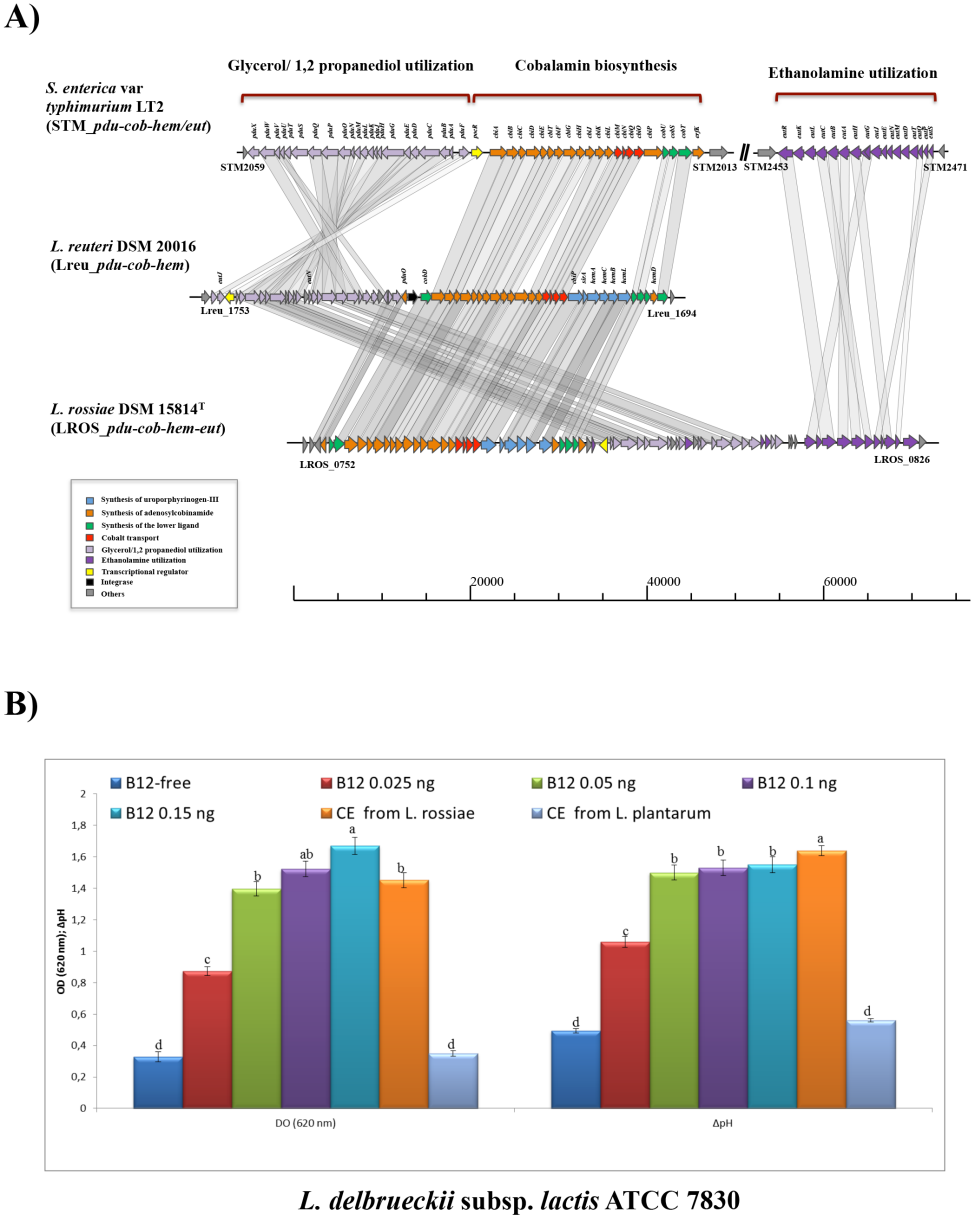
Vitamin B12 production and related ethanolamine utilization. A) Schematic representation of the *pdu-cbi-cob-hem-eth* cluster of *L. rossiae* DSM 15814^T^. Locus map displaying the organization of the *pdu-cbi-cob-hem-eth* of *L. rossiae* DSM 15814^T^ and BLAST-based comparison with the relative cluster of *S. enterica* var Typhimurium LT2 and *L. reuteri* DSM 20016. Synteny map was reconstructed using a combination of reciprocal best BLASTP and CloneManager suite 5 (http://www.scied.com/). B) Growth of *L. delbrueckii* subsp. *lactis* ATCC 7830 in vitamin B12-free medium supplemented with increasing concentrations of vitamin B12 or CE of *L. rossiae* DSM 15814^T^ or *L. plantarum* DC400 as negative control. Optical density (620 nm) and acidification (ΔpH) are also reported.

Among lactobacilli evidence of a *pdu-cbi-cob-hem* cluster has been extensively described in *L. reuteri* DSM 20026, where *cbi-cob-hem* genes are involved in cobalamin production, while the *pdu* locus encodes propanediol and glycerol dehydratase activity which requires B_12_ as a cofactor [23]. In *L. reuteri* DSM 20026 the acquisition of this locus through horizontal transfer has been proposed because of the presence of *IS* elements in its flanking regions, even though the mechanism of acquisition is still unclear [23]. Comparison of the genetic organization of this locus in *L. rossiae* DSM 15814^T^ with that of *L. reuteri* DSM 20026 and the pathogen *Salmonella enterica* var. Typhimurium LT2 showed that gene synteny of these gene clusters is conserved. The only apparent difference is the relative position of the transcriptional regulator *pocR*, which is located at the beginning of the *pdu-cbi-cob-hem* gene cluster in *L. reuteri* (Lreu_1750) [24], while it is located in the middle of the equivalent gene cluster in *S. enterica* and *L. rossiae* (Figure 4A), supporting the hypothesis proposed for *L. reuteri* DSM 20016 that the genetic transfer of the gene cluster occurred by means of two independent events [23]. It is also worth mentioning that the downstream located ethanolamine utilization cluster (*eut*) of DSM 15814^T^ represents a so far unique feature within the genus *Lactobacillus* and that protein products of these genes exhibit 30–40% similarity to the corresponding proteins encoded by genes STM2452-70 of *S. enterica* var. Typhimurium LT2, supporting the notion that horizontal transfer of this region from a progenitor or close relative of *S. enterica* var. Typhimurium may have occurred in *L. rossiae*, and that the two species adopted a similar cobalamin-dependent ethanolamine-propanediol utilization strategy. Previous studies have indicated that ethanolamine utilization is a feature that is not uncommon among gut pathogens and gut commensals (e.g. *Salmonella*, *Enterococcus*, *Pseudomonas*, *Corynebacterium*, *Clostridium*, *Escherichia*) [25]. Phosphatidylethanolamine represents a major component of bacterial and epithelial membranes and may also be present in the gut as a result of the host's diet. For this reason the ability to metabolize this particular compound may confer a distinct advantage to a bacterium in a particular metabolic niche [25]. Cobalamin-dependent utilization of ethanolamine and 1,2-propanediol as a sole carbon source can occur in bacteria under both aerobic and anaerobic conditions [26], while the *de novo* biosynthesis of the cofactor vitamin B_12_ occurs under anaerobic conditions. A recent comparative study conducted on the ethanolamine utilization cluster (*eut*) in bacteria highlighted that the ethanolamine-ammonia lyase-encoding genes (*eutB* and *eutC*) are the most conserved among species (in our case LROS_0819-20), while the accessory genes (e.g., *eutK*, *eutL*, *eutM*, *eutN*, *eutS*), homologues of which appear to be absent from the *L. rossiae* DSM 15814^T^ genome, are believed to be involved in the formation of a metabolosome, yet are not strictly required as they merely seem to increase the substrate utilization efficiency [27].

In order to obtain a phenotypic confirmation of the biosynthetic capacity of B_12_ production in *L. rossiae* DSM 15814^T^ a bioassay was performed using *Lactobacillus delbrueckii* ATCC 7830, a strain known as being unable to grow in the absence of vitamin B_12_ cofactor (see Methods section). During this experiment growth of *L. delbrueckii* ATCC 7830 was observed only following the addition of cytoplasmic extract (CE) obtained from *L. rossiae* or, alternatively, vitamin B_12_ to the medium, thus confirming that *L. rossiae* is capable of producing B_12_ and that this cofactor is present in its CE (Figure 4B). As a negative control we used CE from *L. plantarum* DC400, which as expected failed to support growth of *L. delbrueckii* ATCC 7830.

### B-complex vitamins production in *L. rossiae*

**Figure 5:**
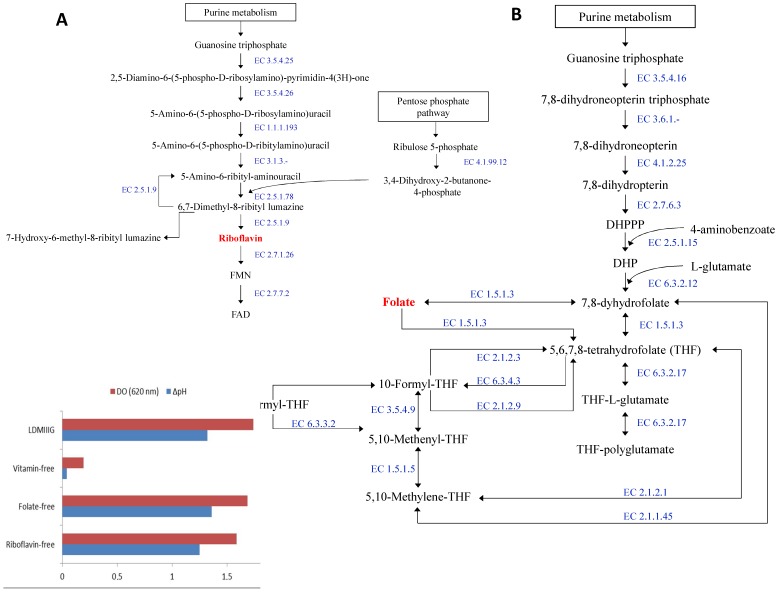
Biosynthesis of riboflavin and folate. Reconstruction of the biosynthesis of riboflavin (A) and folate (B) in *L. rossiae* DSM 15814^T^. Growth and acidification (ΔpH) of DSMZ 15184^T^ at 30°C for 18 h (C). LDMIIIG, modified semi-defined medium LDMIIIG; vitamin-free, LDMIIIG without added vitamins; folate-free, LDMIIIG without folate; riboflavin-free, LDMIIIG without riboflavin. Data are the means of three separate experiments performed in triplicate.

*In silico* analyses of the genome of *L. rossiae* DSM 15814^T^ indicated the potential for *de novo* synthesis of three B group or B-complex vitamins: cobalamin, riboflavin and folate. All these vitamins are important micronutrients, which are involved in a number of vital biochemical reactions of all living cells. Humans do not possess the capacity to synthesize folate, riboflavin, thiamine and cobalamin, and, consequently, they have to obtain such vitamins exogenously. Due to the relevant biotechnological applications, the biosynthetic pathways of B-complex vitamins have been described in detail for food- and gut-associated microorganisms [28]. As shown for members of the genus *Lactobacillus* and *Lactococcus* [24], [29], *L. rossiae* DSM 15814^T^ is predicted to synthesize riboflavin from D-ribulose 5-phosphate and guanosine triphosphate (GTP) (Figure 5A). Indeed, being consistent with this genomic data, *L. rossiae* DSM 15814^T^ was shown to be able to grow in and acidify riboflavin-free LDMIIIG broth (Figure 5C). *L. rossiae* DSM 15814^T^ is also predicted to synthesize folate, but only in the presence of one of its precursors para-aminobenzoic acid (pABA) (Figure 5B). In fact, among the lactobacilli, *L. plantarum* is the only species predicted to be able to synthesize pABA [30]. In support of this *in silico* prediction, *L. rossiae* DSM 15814^T^ growth was shown to occur in folate-free LDMIIIG medium to which pABA had been added (Figure 5C). Nine genes are present in the *L. rossiae* DSM 15814^T^ genome with predicted functions related to thiamine biosynthesis, however, no growth or acidification were observed on thiamine-free LDMIIIG broth (data not shown), indicating that this biosynthetic pathway is incomplete or non-functional.

### Carbohydrate utilization and pyruvate metabolism

*In silico* analyses also predicted that the *L. rossiae* DSM 15814^T^ genome harbours a substantial number of genes involved in carbohydrate utilization. Based on previous studies, DSM 15814^T^ is capable of metabolizing ribose, D-xylose, arabinose, galactose, glucose, fructose, mannose, N-acetyl glucosamine, maltose, turanose, D-gluconic acid, palatinose, α-hydroxy-butyric acid and D-L lactic acids, when grown in MRS medium [1], [16]. Bioinformatic analyses of the DSM 15814^T^ genome sequence revealed 17 complete phosphoenolpyruvate phospho-transferase systems (PEP-PTS) belonging to different families (e.g., Glc, Lac and Man families), but also putative ABC transporters involved in the uptake of various carbohydrates (e.g maltose/maltodextrin, ribose, fucose, glucose, galactose, lactose, galactose and N-acetyl-D-glucosamine).

In addition, *L. rossiae* DSM 15814^T^ is also predicted to produce a number of extracellular or cell wall-associated polysaccharide-degrading enzymes, represented by a cyclomaltodextrinase (E.C. 3.2.1.54; LROS_1707), an α-amylase (E.C. 3.2.1.10; LROS_1584), a β-glucosidase (E.C. 3.2.1.21; LROS_2047), a mannosyl-glycoprotein endo-beta-N-acetylglucosaminidase (E.C. 3.2.1.96; LROS_0612) and a neopullulanase (EC 3.2.1.135; LROS_1707). Furthermore, the *L. rossiae* DSM 15814^T^ genome specifies putative enzymatic pathways predicted to degrade arabinose and xylose-containing poly- and/or oligosaccharides (Figure S2), both of which occur as major carbohydrate components in plant cells. In particular two genes (LROS_1251 and LROS_1108) are present which encode predicted extracellular glycosyl hydrolases responsible for the initial degradation of arabinose and xylose-containing polysaccharides into oligosaccharides prior to their internalization, after which they are further hydrolysed to xylose/arabinose.

Regarding pyruvate metabolism, both D- and L-lactate dehydrogenase-encoding genes (*ldhD*: LROS_0202, LROS_0465, LROS_2176, LROS_2251 and *ldhL*: LROS_1559, LROS_2223, LROS_2704) are present, which convert pyruvate into D- and L-lactate, respectively (Figure S3). The presence of multiple copies of *ldh* genes is not a peculiarity of *L. rossiae* as this has also been found in other lactobacilli, possessing both large (e.g., *L. plantarum*, *L. casei*) or small (e.g., *L. sanfranciscensis*) genomes [30], [31], [32]. The same can be said for genes whose encoded enzymes are predicted to lead to the synthesis of ethanol and acetic acid from pyruvate, or catabolize pyruvate to other compounds such as acetaldehyde and acetoin.

Furthermore, it has previously been demonstrated that growth of hetero-fermentative lactic acid bacteria is enhanced when certain organic carbon sources (for example citrate) are used as external electron acceptors [33], a notion enforced by the fact that *L. rossiae* DSM 15814^T^ encompasses several genes with predicted functions in citrate metabolism (LROS_0055-LROS_0059).

### Amino acid biosynthesis, catabolism and proteolytic system

The various biosynthetic pathways involved in amino acid synthesis are variably present or absent in the different members of the *Lactobacillus* genus [12], [32] and in this regard *L. rossiae* DSM 15814^T^ is predicted to contain genes encoding the enzymes that make up biosynthetic pathways for certain amino acids (e.g. serine, glycine, L-asparagine, glutamine, L-cysteine and L-methionine).

Regarding the catabolism of free amino acids, the *L. rossiae* DSM 15814^T^ genome is predicted to specify a complete arginine deaminase (ADI) pathway, glutamine synthase and glutamate decarboxylase enzymes (Table S3, Figure S4 and S5 and Text S1).

The ADI gene cluster, characterized in lactococci and lactobacilli, has been correlated with having a positive effect on the flavour of leavened baked goods [33], but is mainly used by the cell as an acid-stress response mechanism through the production of the alkaline compound NH_3_. In addition, the energy (ATP) produced via this pathway enables the extrusion of cytoplasmic protons through the F_0_F_1_ ATPase, thus supporting cell survival following sugar depletion [34]. Activity of the ADI pathway in *L. rossiae* DSM 15814^T^ was detected at phenotypic level (Figure S5) by determining the kinetics of growth and acidification with and without arginine supplementation in the medium. Compared to the negative control (e.g. medium without arginine addition), cell density of *L. rossiae* DSM 15814^T^ did not differ (P>0.05) in the arginine-supplemented medium. However, an increase of cell survival was found after 48 h in the growth medium containing arginine compared to the same medium without arginine. Due to the production of NH_3_ from arginine metabolism, different kinetics of acidification were found, which at 24 h showed a substantial difference of pH 6.79 (arginine-containing medium) versus 4.32 (medium without arginine). The activity of all three ADI pathway enzymes was also found in the cytoplasmic extract of DSM 15814^T^ (80, 140, and 87 U/mg for ADI, OTC, and CK, respectively). Similar data was previously found for *L. sanfranciscensis* [34].

Glutamate is inter-convertible to glutamine through the activity of the glutamine synthase (GS, EC 6.3.1.2). The genome of *L. rossiae* contains a gene that is predicted to encode a glutamate decarboxylase (EC 4.1.1.15), which converts glutamate into γ-amino butyric acid (GABA). In addition, the production of GABA was also confirmed phenotypically. The capacity for synthesizing GABA of *L. rossiae* was assayed in SDB medium. After 24 h of fermentation at 30°C, the concentration of GABA was 1.75 mg/kg. GABA, a four-carbon non-protein amino acid, has been reported to act as the major inhibitory neurotransmitter of the central nervous system, to possess anti-hypertensive, diuretic and tranquilizer effects, and to prevent diabetes [35].

Except for two putative and uncharacterized zinc proteases (metalloproteases) (LROS_1355; LROS_2169), no predicted extracellular proteinase (*prt*) genes were found in the genome of *L. rossiae*, which is in agreement with the fact that *L. rossiae* was shown to be unable to grow in milk without the addition of peptides or free amino acids. In addition, only a weak protease activity was found on albumin and globulin (data no shown).

As shown for *L. sanfranciscensis* [32], [36], *L. rossiae* has more than 20 genes encoding putative intracellular peptidases with different specificities (e.g. aminopeptidase and oligo-/tri-/di-peptidase activities), which may confer this strain the ability of hydrolysing a broad range of peptides (Table S4). In support of this *in silico* prediction, *L. rossiae* DSM 15814^T^ showed peptidase activities (general aminopeptidase type N, PepN; proline iminopeptidase, PepI; X-prolyl dipeptidyl aminopeptidase, PepX; endopeptidase, PepO; prolyl endopeptidyl peptidase, PEP; tripeptidase, PepT; dipeptidase, PepV; prolidase, PepQ; and prolinase, PepR (Figure S6). Peptidase activities improve the organoleptic characteristics of fermented foods (e.g., sourdough leavened baked goods) by increasing the concentration of free amino acids [33].

### Regulation of *L. rossiae*

Regulatory proteins play an important role in the adaptation of bacteria to different environments and *L. rossiae* DSM 15814^T^ encodes quite a high number of putative regulatory genes (117, representing 4.3% of the total identified protein complement), some of which are involved in stress response (Table S5). The number of transcriptional regulators of *L. rossiae* is markedly higher than that found for *L. delbrueckii* subsp. *bulgaricus* (ca. 53, representing 3.1% of total of gene content), *L. helveticus* (ca. 44, representing 2.7% of the total of gene content) or *L. sanfranciscensis* (ca. 40, representing 2.8% of the total of gene content), while it is similar to that found for the highly versatile species *L. casei* (ca. 140, representing 4.5% of the total of gene content), yet lower than *L. plantarum* (ca. 234, representing 7.7% of the total of genes). These findings are in agreement with the notion that large genomes specify a relative high number of regulatory proteins [37], [12].

## Conclusions

Since its isolation, *L. rossiae* has been shown to be a highly versatile species capable of colonizing different environments, such as fermented cereals, legumes, fruits and meat, as well as being an inhabitant of the human and animal gut-intestinal tract. Analysis of *L. rossiae* DSM 15814^T^, a sourdough strain, revealed a minimum predicted genome size of 2,870,031 bp, encoding relatively high numbers of genes involved in carbohydrate and amino acid transport and metabolism, stress resistance, transcriptional regulation and signal transduction. Based on phylogeny, phylogenomic and comparative analyses, the *L. rossiae* taxon forms an independent phylogenetic branch, in close proximity to the *L. reuteri, L. fermentum*, *L. plantarum* and *L. brevis* groups, which suggests that these species all adopted a similar strategy for phylogenetic evolution and environmental adaptation. From a technological point of view, the genome of *L. rossiae* DSM 15814^T^ shows some interesting features: (i) the *de novo* production of B_12_ cofactor and other B-complex vitamins (e.g. riboflavin and folate), which represents an important biotechnological potential for industrial applications; (ii) the synthesis of flavor-related compounds from the pyruvate metabolism, proteolysis and amino acid catabolism; and (iii) the capacity to use a substantial number of carbon sources, which provides metabolic flexibility in terms of nutrient availability. In addition, the high number of genes correlated to regulatory systems indicates that *L. rossiae* is a versatile species with a high potential for environmental colonization. The results of this study are expected to provide the foundation for further investigations to exploit the biotechnology potential of *L. rossiae* isolates.

## Materials and Methods

### Genome annotation

The genome sequence of *L. rossiae* DSM 15814^T^ was obftained from the NCBI database (accession number: NZ_AKZK00000000) and the analysis focused on 57 large contigs (400 bp size cut-off). Open Reading Frame (ORF) prediction and functional assignment was automatically performed using the RAST webserver (http://rast.nmpdr.org/), and the generated information was integrated and assessed with the contribution of alternative databases as protein family (Pfam) (http://pfam.sanger.ac.uk), ENZYME database [38] and Clusters of Orthologous Groups (COGs). In particular, the annotation of the EC numbers of each ORF was carried out using the annotat8r tool [39]. COG classification was carried out using BLASTP alignment against the relative database (http://www.ncbi.nlm.nih.gov/Ftp/databases/COG), using a cut-off E-value of 0.0001 with at least 50% of identity across a minimum of 50% of a given protein length.

### Comparative analyses

BLAST-based comparative analyses at protein level were performed on 20 representative *Lactobacillus* species, including *L. rossiae* DSM 15814^T^, through the bi-directional BLASTP alignment [40], and employing a cut-off E-value of 0.0001, with at least 50% of identity across a minimum of 50% of protein length.

Using Markov Cluster Algorithm (MCL) [41], all identified genes were grouped in orthologous families and the resulting output was used to build a binary matrix containing information on the presence/absence of such families in each *Lactobacillus* species.

Hierarchical clustering analysis was performed with the hclust package in R (http://www.r-project.org), using as input the binary matrix computed with the above method. A heatmap displaying the clustering of organisms and related families was generated through the Multiple Experiment Viewer (MeV) tool v.4.8.1 (http://mev-tm4.sourceforge.net).

### Phylogenomics of *L. rossiae* DSM 15814^T^

First, phylogenetic analysis of *L. rossiae* DSM 15814^T^ and 20 complete *Lactobacillus* genomes was inferred through 16S rDNA alignment in MUSCLE v6.0 [42] and single gene neighbour-joining tree in MEGA6 [43]. As an additional analysis, a multi-locus supertree was computed from the alignment of three concatenated housekeeping genes (*groEL*, *dnaK* and *grpE*) and 96 selected orthologues. The relative phylogenetic relations were inferred using the JTT model in Phyml v3.0 [44]. A consensus tree was computed using the Consense module in Phylip package v3.69 (http://evolution.genetics.washington.edu/phylip.html). In all cases, bootstrap assessment of the inferred relations was carried out by means of 1000 bootstrap iterations.

### Reconstruction of metabolic pathways

Metabolic reconstruction may provide insights regarding the metabolic potential of *L. rossiae* DSM 15814^T^, which should guide experimental studies to investigate genotype–phenotype relationships. EC numbers were extracted from genome annotations. They were then automatically mapped onto the Kyoto Encyclopedia of Genes and Genomes (KEGG) metabolic pathways [45]. In cases where key enzymes were missing within an otherwise complete pathway, a further effort was made to identify homologous candidate enzymes through extensive manual searches with BLASTP and HMMER [46], [37].

### Biosynthesis of riboflavin, folate, thiamine and cobalamin

Growth of *L. rossiae* DSM 15814^T^ was assayed in modified semi-defined medium (LDMIIIG) (pH 5.6) [47], which was supplemented with 0.1% Tween 80 and 1% of peptone (see Text S2).

### Cobalamin detection and quantitative bioassay

To demonstrate cobalamin production by *L. rossiae* DSM 15814^T^, a bioassay was performed using *Lactobacillus leichmannii* ATCC 7830 (formerly *Lactobacillus delbrueckii* subsp. *lactis* ATCC 7830), a strain that requires B_12_ for growth (see Text S2).

### Protease and peptidase activities

Proteinase activity was measured using wheat albumins and globulins as substrates. The level of protein hydrolysis was estimated by Reversed-phase high-performance liquid chromatography (RP-HPLC) and amino acid analyses (see Text S2).

### Arginine catabolism assay, cell survival and biosynthesis of GABA from glutamic acid

*L. rossiae* DSM 15814^T^ was propagated for 24 h at 30°C in modified MRS broth (Oxoid, Basingstoke, Hampshire, England) with the addition of fresh yeast extract (5%, vol/vol) and 28 mM maltose to a final pH of approximately 5.6 [34]; see Text S2). The biosynthesis of GABA from glutamic acid was estimated as described by Siragusa *et al*. [48] using a Biochrom 30 series Amino Acid Analyzer (Biochrom Ltd., Cambridge Science Park, England).

## Supporting Information

Figure S1 **Predicted plasmid of** ***L. rossiae*** **DSM 15814^T^.** Genome atlas representing the organization of ORFs in the predicted plasmid pLR1 of *L. rossiae* DSM 15814^T^. Displayed are, from inner to outer circle: G+C skew, G+C content and ORFs organization. Each ORF is also classified based on the predicted function.
(TIF)

Figure S2 **Utilization of arabinose and xylose-containing poly/oligosaccharides.**
(TIF)

Figure S3 **Pyruvate metabolic pathway of** ***L. rossiae*** **DSM 15814^T^.**
(TIF)

Figure S4 **Locus map showing the organization of the arginine deaminase (ADI) cluster.** *L. rossiae* DSM 15814^T^, *L. amylovorus* GRL 1112, *L. acidophilus* 30SC, *L. fermentum* IFO 3956, *L. sakei* subsp. *sakei* 23K, *L. brevis* ATCC 367, *L. buchneri* NRRL B-30929, *L. kefiranofaciens* ZW3.
(TIF)

Figure S5 **Kinetics of growth (Log CFU/ml) (A) and pH of growth medium (B).** *L. rossiae* DSM 15814^T^ cells were cultivated in MAM broth with (empty triangles) and without (filled triangles) 17 mM arginine.
(TIF)

Figure S6 **Peptidase activities of** ***L. rossiae*** **DSM 15814^T^.**
(TIF)

Table S1 **Selected contig and accession number of the genome of** ***Lactobacillus rossiae*** **DSM 15814^T^.**
(XLSX)

Table S2 **General features of representatives of the genus** ***Lactobacillus*** **analysed in this study.**
(DOCX)

Table S3 ***In silico*** **search of the genome of** ***Lactobacillus rossiae*** **DSM 15814^T^ for ORFs putatively involved in amino acid metabolism.**
(DOCX)

Table S4 ***In silico*** **analysis of the genome of** ***Lactobacillus rossiae*** **DSM 15814^T^ for ORFs encoding proteases and peptidases, and their cleavage specificities, gene names and relative abundance in the genome.**
(DOCX)

Table S5 **Predicted transcriptional regulators encoded by the** ***Lactobacillus rossiae*** **DSM 15814^T^ genome.**
(DOCX)

Text S1 **Results - Supporting Information.**
(DOCX)

Text S2 **Experimental Procedures - Supporting Information.**
(DOCX)
