# Supplementary figures and images for: Lactobacillus rossiae, a Vitamin B12 Producer, Represents a Metabolically Versatile Species within the Genus Lactobacillus
Source: PLoS One. 2014 Sep 29;9(9):e107232. doi: 10.1371/journal.pone.0107232 (PMC4180280; doi:10.1371/journal.pone.0107232)

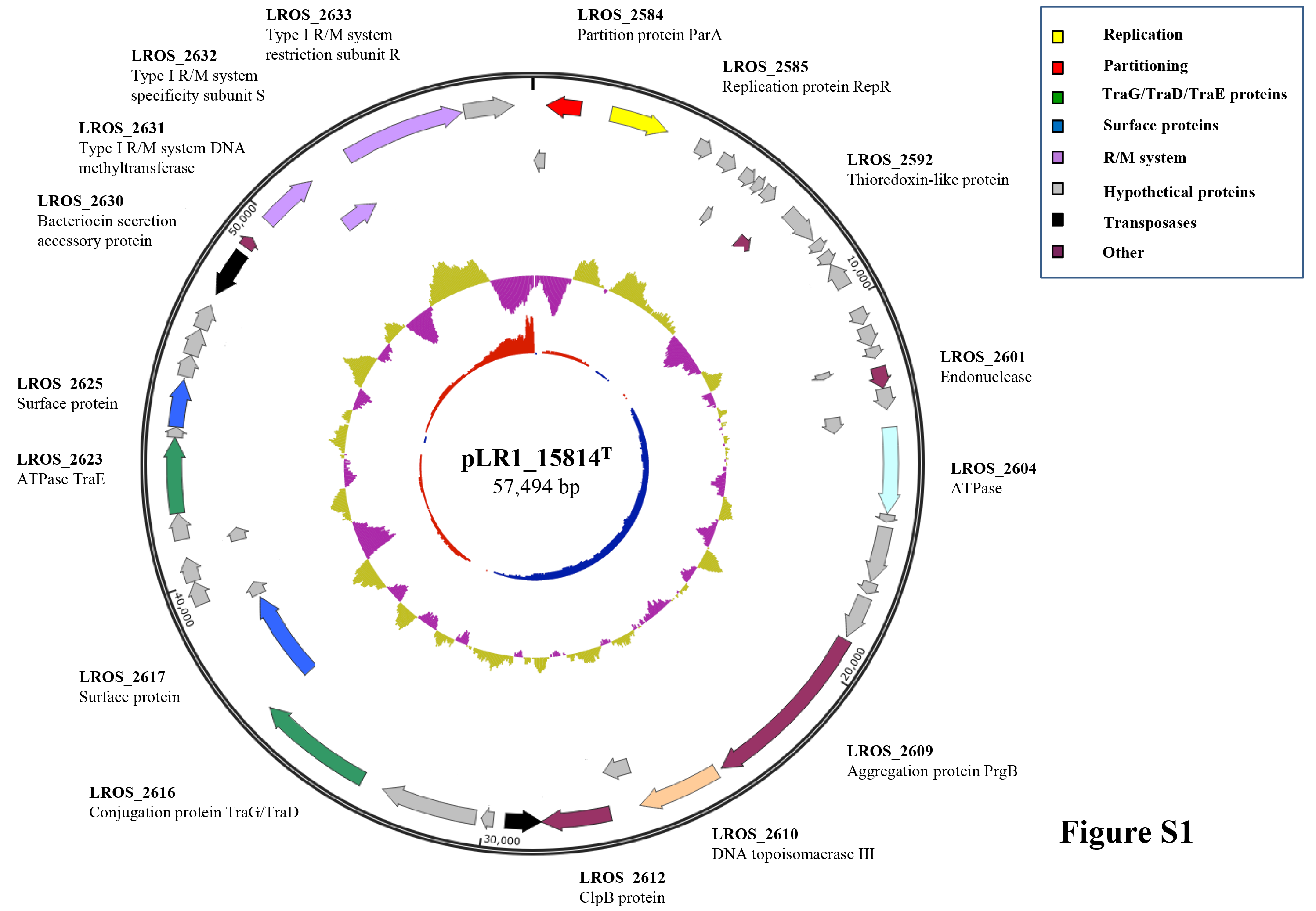

Supplement: Figure S1 — Predicted plasmid of L. rossiae DSM 15814T. Genome atlas representing the organization of ORFs in the predicted plasmid pLR1 of L. rossiae DSM 15814T. Displayed are, from inner to outer circle: G+C skew, G+C content and ORFs organization. Each ORF is also classified based on the predicted function. (TIF) [file pone.0107232.s001.tif]

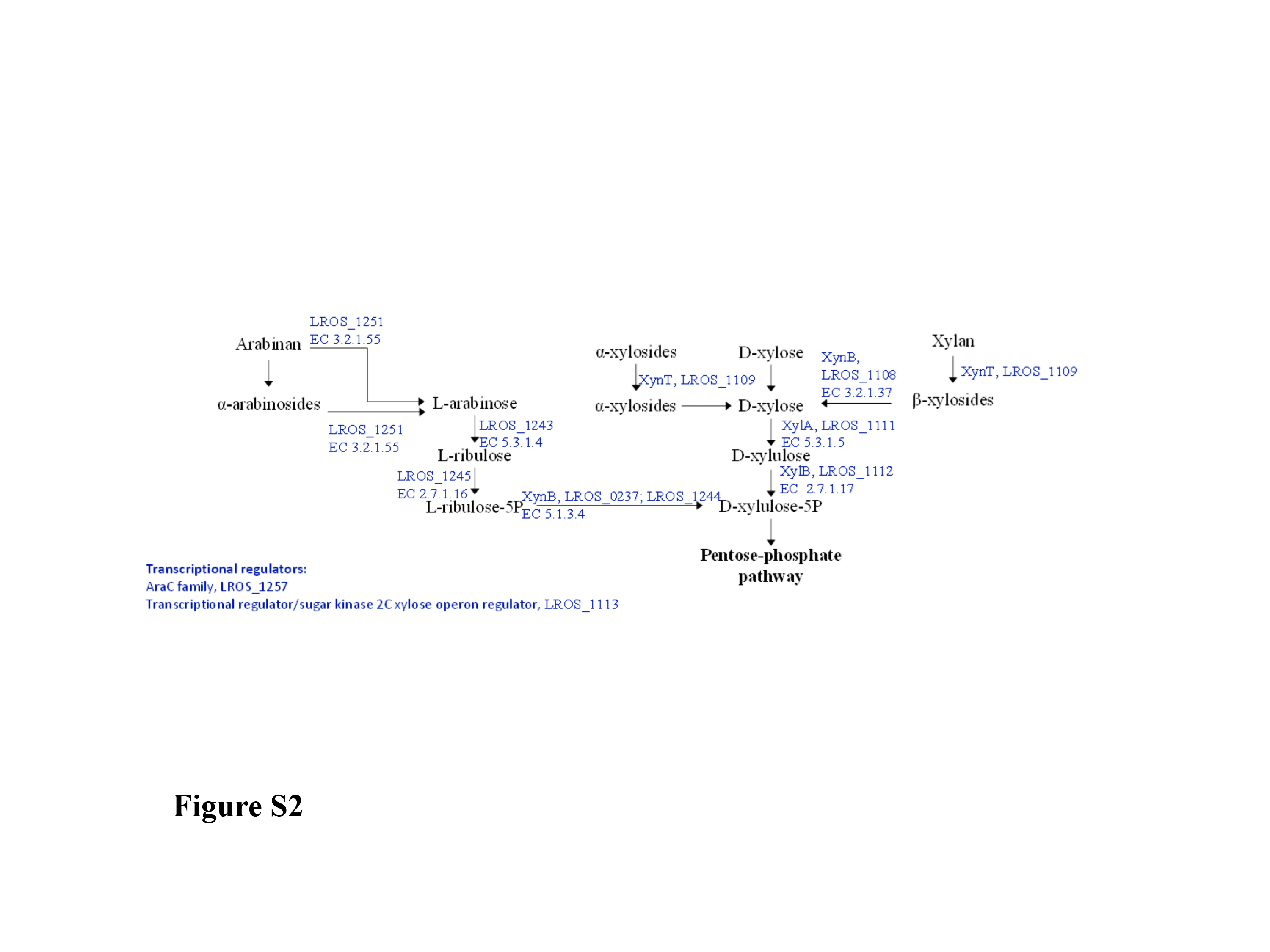

Supplement: Figure S2 — Utilization of arabinose and xylose-containing poly/oligosaccharides. (TIF) [file pone.0107232.s002.tif]

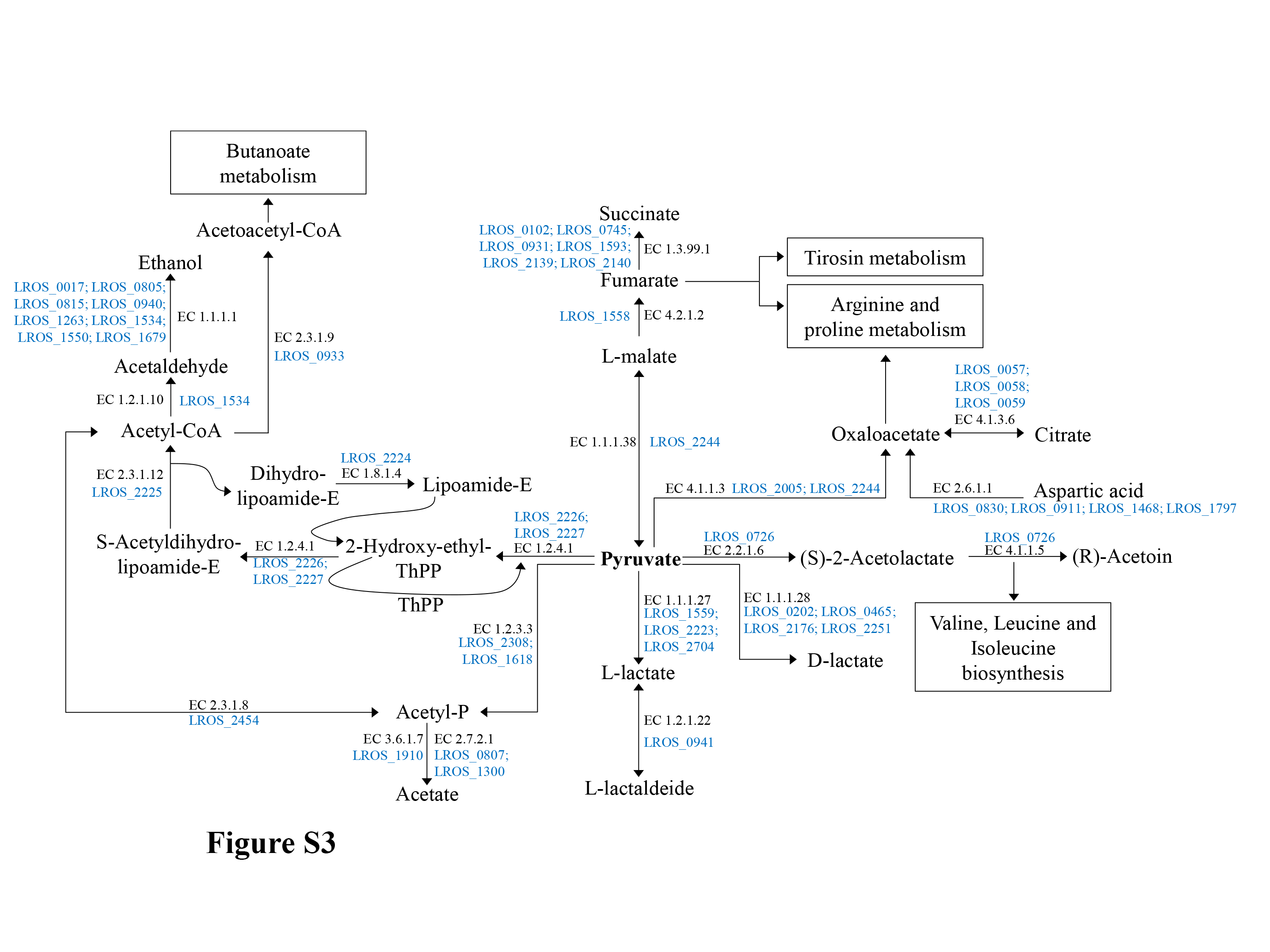

Supplement: Figure S3 — Pyruvate metabolic pathway of L. rossiae DSM 15814T. (TIF) [file pone.0107232.s003.tif]

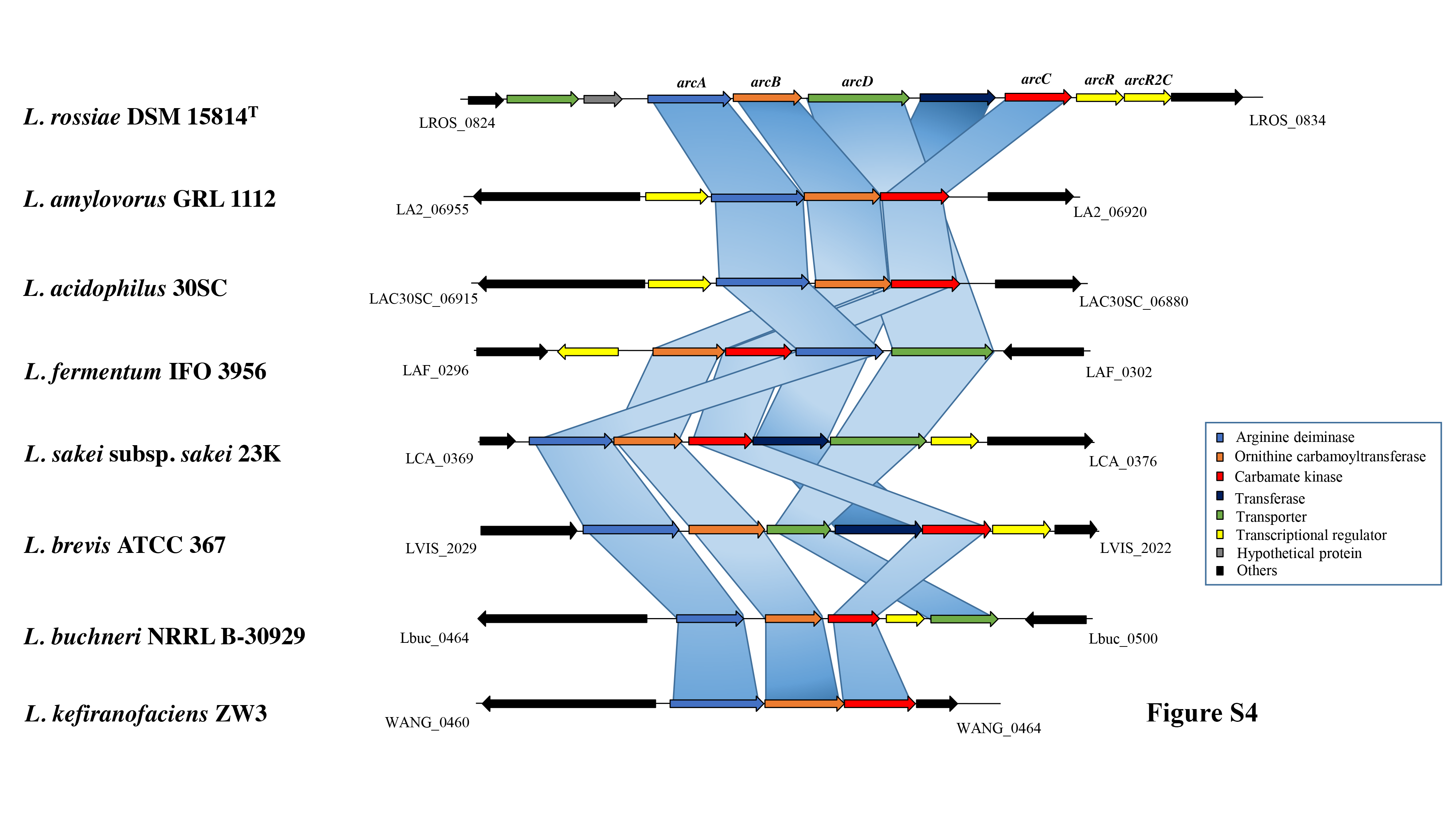

Supplement: Figure S4 — Locus map showing the organization of the arginine deaminase (ADI) cluster. L. rossiae DSM 15814T, L. amylovorus GRL 1112, L. acidophilus 30SC, L. fermentum IFO 3956, L. sakei subsp. sakei 23K, L. brevis ATCC 367, L. buchneri NRRL B-30929, L. kefiranofaciens ZW3. (TIF) [file pone.0107232.s004.tif]

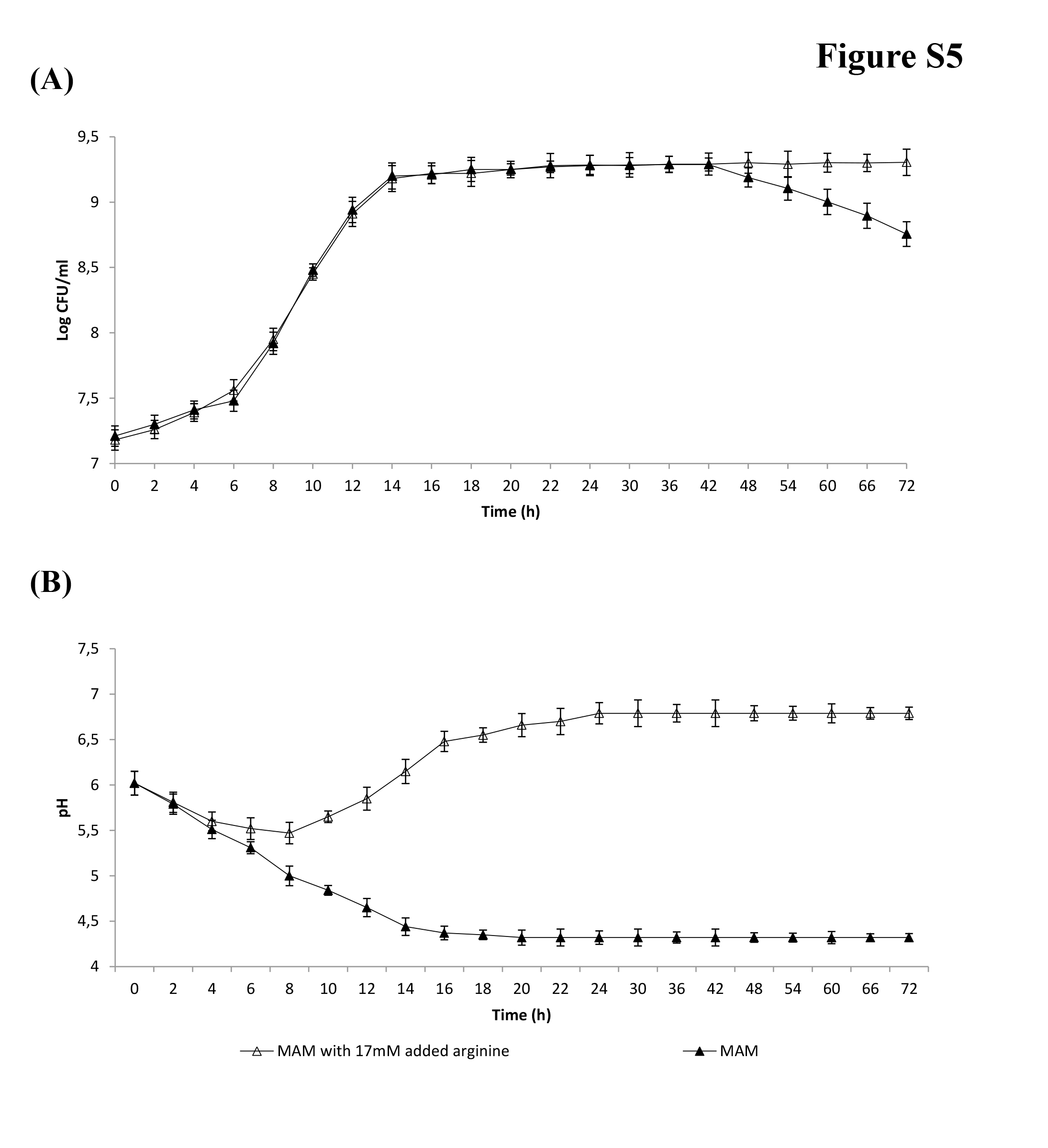

Supplement: Figure S5 — Kinetics of growth (Log CFU/ml) (A) and pH of growth medium (B). L. rossiae DSM 15814T cells were cultivated in MAM broth with (empty triangles) and without (filled triangles) 17 mM arginine. (TIF) [file pone.0107232.s005.tif]

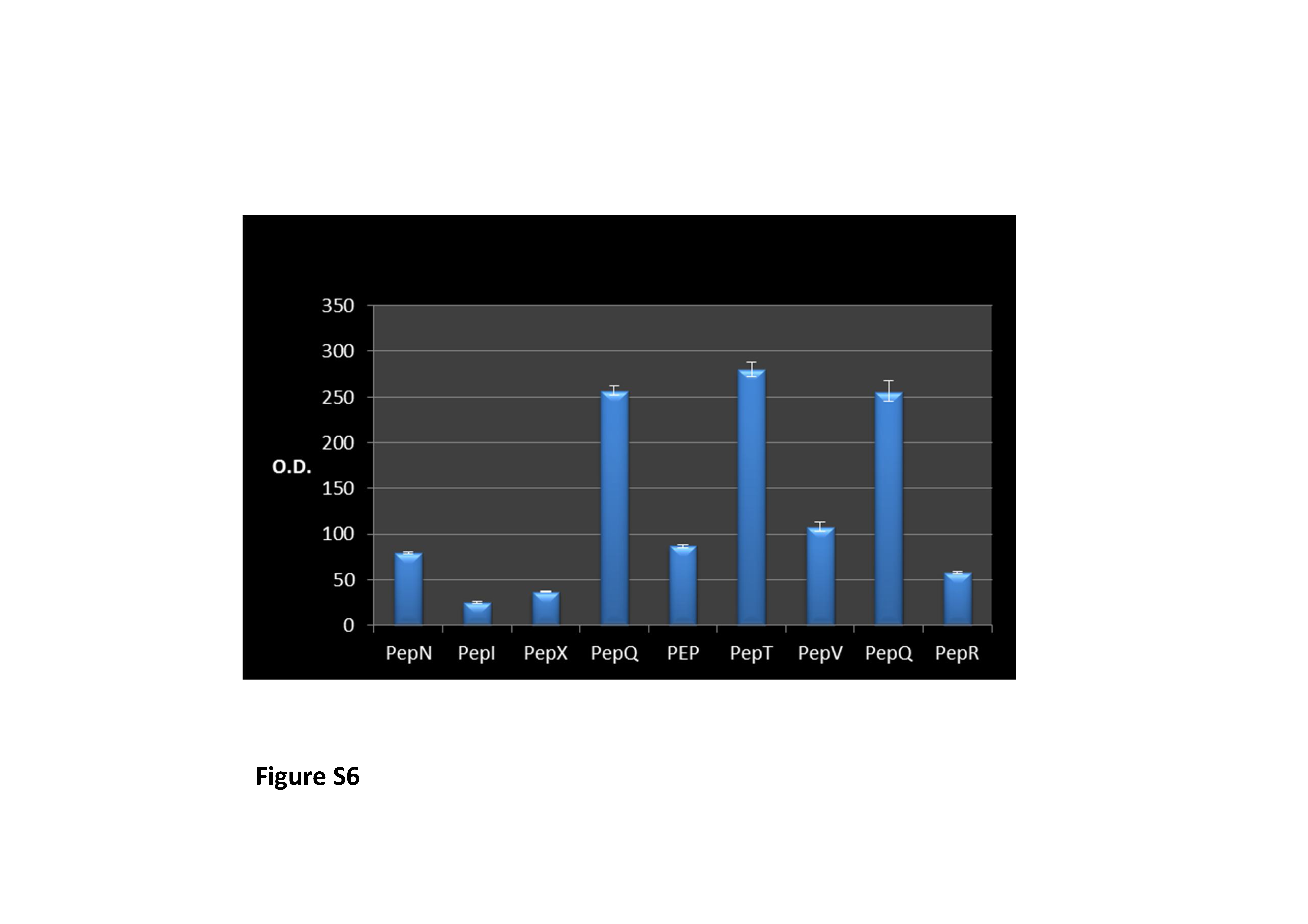

Supplement: Figure S6 — Peptidase activities of L. rossiae DSM 15814T. (TIF) [file pone.0107232.s006.tif]
