# Supplementary material for: Lactobacillus rossiae, a Vitamin B12 Producer, Represents a Metabolically Versatile Species within the Genus Lactobacillus
Source: PLoS One. 2014 Sep 29;9(9):e107232. doi: 10.1371/journal.pone.0107232 (PMC4180280; doi:10.1371/journal.pone.0107232)
Supplement: Table S2 — General features of representatives of the genus Lactobacillus analysed in this study. (DOCX) [file pone.0107232.s008.docx]

**Table S2.** General features of representatives of the genus *Lactobacillus* analysed in this study.

| **Name** | **Genome size (Mb)** | **ORFs** | **Origin** | **G+C mol %** |
| --- | --- | --- | --- | --- |
| ***L. rossiae* DSM 15814^T^** | 2.87 | 2,701 | Sourdough | 43.5 |
| ***L. gasseri* ATCC 33323** | 1.89 | 1,755 | Human isolate | 35.3 |
| ***L. johnsonii* NCC 533** | 1.99 | 1,821 | Human isolate | 34.5 |
| ***L. delbrueckii* subsp. *bulgaricus* ATCC 11842** | 1.87 | 1,529 | Fermented milk | 49.7 |
| ***L. helveticus* DPC 4571** | 2.08 | 1,610 | Cheese isolate | 37.1 |
| ***L. amylovorus* GRL 1112** | 2.13 | 2,121 | Porcine faeces | 38.1 |
| ***L. crispatus* ST1** | 2.04 | 2,021 | Chicken’s crop | 36.9 |
| ***L. kefiranofaciens* ZW3** | 2.35 | 2,162 | Kefir isolate | 37.4 |
| ***L. casei* ATCC334** | 2.92 | 2,922 | Cheese isolate | 46.6 |
| ***L. paracasei* subsp. *paracasei* 87002** | 3.03 | 2,890 | Human gut | 46.3 |
| ***L. rhamnosus* ATCC 8530** | 2.96 | 2,886 | Human gut | 46.8 |
| ***L. plantarum* WCFS1** | 3.35 | 3,063 | Human saliva | 44.5 |
| ***L. brevis* ATCC 367** | 2.34 | 2,218 | Human gut | 46 |
| ***L. buchneri* NRRL B-30929** | 2.59 | 2,392 | Ethanol production facility | 44.2 |
| ***L. fermentum* IFO 3956** | 2.1 | 1,843 | Fermented plant material | 51.5 |
| ***L. reuteri* DSM 20016** | 2.09 | 2,054 | Human isolate | 38.9 |
| ***L. sakei* subsp. *sakei* 23K** | 1.88 | 1,871 | Meat | 41.3 |
| ***L. ruminis* ATCC 27782** | 2.07 | 1,862 | Bovine isolate | 43.5 |
| ***L. salivarius* UCC118** | 2.13 | 2,013 | Human gut | 33 |
| ***L. acidophilus* 30SC** | 2.1 | 2,059 | Swine intestine | 38.1 |
| ***L. sanfranciscensis* ATCC 27651** | 1.38 | 1,284 | Sourdough | 34.8 |
