# Supplementary material for: Lactobacillus rossiae, a Vitamin B12 Producer, Represents a Metabolically Versatile Species within the Genus Lactobacillus
Source: PLoS One. 2014 Sep 29;9(9):e107232. doi: 10.1371/journal.pone.0107232 (PMC4180280; doi:10.1371/journal.pone.0107232)
Supplement: Table S3 — In silico search of the genome of Lactobacillus rossiae DSM 15814T for ORFs putatively involved in amino acid metabolism. (DOCX) [file pone.0107232.s009.docx]

**Table S3**. *In silico* search of the genome of *Lactobacillus rossiae* DSM 15814^T^ for ORFs putatively involved in amino acid metabolism.

| **Amino acid** | **Gene** | **No. of genes** | **Locus number** |
| --- | --- | --- | --- |
| Arginine | Arginine/ornithine antiporter ArcD | 3 | LROS_0825; LROS_0829; LROS_2255 |
|  | Arginine deiminase (EC 3.5.3.6) | 1 | LROS_0827 |
|  | Ornithine carbamoyltransferase (EC 2.1.3.3) | 1 | LROS_0828 |
|  | Carbamate kinase (EC 2.7.2.2) | 1 | LROS_0831; LROS_2282 |
|  | Arginine pathway regulatory protein ArgR2C repressor of arg regulon | 2 | LROS_0833; LROS_1817 |
|  | Arginine catabolic regulator | 1 | LROS_1950 |
|  | Ornithine decarboxylase (EC 4.1.1.17) | 2 | LROS_2257; LROS_2265 |
|  | Putrescine/proton symporter putrescine/ornithine antiporter PotE | 1 | LROS_2264 |
| Aspartate | Aspartate aminotransferase (EC 2.6.1.1) | 3 | LROS_0830; LROS_0911; LROS_1468; LROS_1797 |
|  | Aspartokinase (EC 2.7.2.4) | 1 | LROS_0904 |
|  | Aspartate-semialdehyde dehydrogenase (EC 1.2.1.11) | 1 | LROS_0912 |
|  | Aspartate carbamoyltransferase (EC 2.1.3.2) | 1 | LROS_1638 |
| Glutamate | Proton/glutamate symport protein - Sodium/glutamate symport protein | 1 | LROS_0200 |
|  | Glutamate decarboxylase (EC 4.1.1.15) | 1 | LROS_0518 |
|  | Glutamyl-tRNA synthetase (EC 6.1.1.17) Glutamyl-tRNA(Gln) synthetase (EC 6.1.1.24) | 1 | LROS_0519 |
|  | Probable glutamate/gamma-aminobutyrate antiporter | 1 | LROS_0279 |
|  | Glutamate-1-semialdehyde aminotransferase (EC 5.4.3.8) | 1 | LROS_0780 |
|  | Glutamyl endopeptidase precursor (EC 3.4.21.19) (Staphylococcal serine proteinase) (V8 protease) (V8 proteinase) (Endoproteinase Glu-C) | 1 | LROS_1134 |
|  | Glutamate racemase (EC 5.1.1.3) | 1 | LROS_1320 |
|  | Glutamate transport ATP-binding protein | 1 | LROS_1734 |
|  | Glutamate transport substrate-binding protein | 1 | LROS_1735 |
|  | Glutamate transport membrane-spanning protein | 2 | LROS_1736; LROS_1737 |
| Glutamine | Glutamine amidotransferase class I | 1 | LROS_1384 |
|  | Glutamine ABC transporter ATP-binding protein | 1 | LROS_2199 |
| Serine | Serine O-acetyltransferase (EC 2.3.1.30 ) | 2 | LROS_0271; LROS_0342 |
| **Amino acid** | **Gene** | **No. of genes** | **Locus number** |
| Serine | Putative serine/tyrosine protein phosphatase( EC 3.1.3.48) | 1 | LROS_0375 |
|  | D-serine/D-alanine/glycine transporter | 1 | LROS_1080; LROS_1299; LROS_1804 |
|  | Serine hydroxymethyltransferase (EC 2.1.2.1) | 1 | LROS_1088 |
|  | Serine acetyltransferase (EC 2.3.1.30) | 1 | LROS_0342 |
| Cysteine | Cysteine synthase (EC 2.5.1.47) | 1 | LROS_0343 |
|  | Cysteine desulfurase (EC 2.8.1.7) | 2 | LROS_0708; LROS_1370 |
|  | Cystine transport system permease protein | 1 | LROS_1590 |
| Methionine | Methionine ABC transporter substrate-binding protein | 1 | LROS_0717 |
|  | Methionine ABC transporter ATP-binding protein | 1 | LROS_0718 |
|  | Methionine ABC transporter permease protein | 1 | LROS_0719 |
|  | Free methionine-(R)-sulfoxide reductase contains GAF domain | 1 | LROS_1372 |
|  | Peptide methionine sulfoxide reductase MsrA (EC 1.8.4.11) | 1 | LROS_2061 |
|  | Peptide methionine sulfoxide reductase MsrB (EC 1.8.4.12) | 1 | LROS_2062 |
| Glycine | Glycine cleavage system H protein (lipoate-binding) | 1 | LROS_1385 |
