# Supplementary material for: Lactobacillus rossiae, a Vitamin B12 Producer, Represents a Metabolically Versatile Species within the Genus Lactobacillus
Source: PLoS One. 2014 Sep 29;9(9):e107232. doi: 10.1371/journal.pone.0107232 (PMC4180280; doi:10.1371/journal.pone.0107232)
Supplement: Table S4 — In silico analysis of the genome of Lactobacillus rossiae DSM 15814T for ORFs encoding proteases and peptidases, and their cleavage specificities, gene names and relative abundance in the genome. (DOCX) [file pone.0107232.s010.docx]

**Table S4.** *In silico* analysis of the genome of *Lactobacillus rossiae* DSM 15814^T^ for ORFs encoding proteases and peptidases, and their cleavage specificities, gene names and relative abundance in the genome.

| **Enzyme** | **Gene** | **No. of genes** | **Locus number** |
| --- | --- | --- | --- |
| **Protease** | Putative metallopeptidase (Zinc) SprT family | 1 | LROS_0426 |
|  | Predicted Zn-dependent protease | 1 | LROS_0943 |
| **Endopeptidases** | Endopeptidase (*pepO,*  EC= 3.4.24-) | 2 | LROS_2665 |
|  | oligoendopeptidase F (*pepF*  EC= 3.4.24-) | 4 | LROS_0642; LROS_1022;  LROS_1023; LROS_1484 |
| **Aminopeptidases** | Aminopeptidase C (*pepC*, EC 3.4.22.40) | 2 | LROS_0449; LROS_1166 |
|  | Lysyl aminopeptidase (EC 3.4.11.15) | 1 | LROS_0368 |
|  | Proline iminopeptidase ( *pepI*, EC 3.4.11.5) | 2 | LROS_1191; LROS_2679 |
|  | Methionine aminopeptidase ( *MAP*, EC 3.4.11.18) | 1 | LROS_1568 |
|  | Aminopeptidase YpdF | 1 | LROS_1941 |
| **Oligo-/Tri-/di-peptidases** | Prolyl-dipeptidase (prolinase, *pepR*) | 1 | LROS_1313 |
|  | Tripeptide aminopeptidase ( *pepT*, EC 3.4.11.4) | 1 | LROS_2121 |
|  | Xaa-Pro dipeptidyl-peptidase (*pepX*, EC) | 1 | LROS_2689 |
|  | Xaa-His dipeptidase (EC 3.4.13.3) | 1 | LROS_1771 |
|  | Dipeptidase (*pepV*, EC 3.4.13.18) | 2 | LROS_0363; LROS_1996 |
|  | Pyrrolidone-carboxylate peptidase (EC 3.4.19.3) | 1 |  |
|  | D-alanyl-D-alanine carboxypeptidase (EC 3.4.16.4) | 1 | LROS_1754 |
| **Uncharacterized** | M16 family peptidase | 1 | LROS_1354 |
|  | Dipeptidase | 1 | LROS_0333 |
|  | Dipeptidase | 2 | LROS_0477; LROS_2668 |
|  | Peptidyl-prolyl cis-trans isomerase (EC 5.2.1.8) | 1 | LROS_1293 |
