# Supplementary material for: Lactobacillus rossiae, a Vitamin B12 Producer, Represents a Metabolically Versatile Species within the Genus Lactobacillus
Source: PLoS One. 2014 Sep 29;9(9):e107232. doi: 10.1371/journal.pone.0107232 (PMC4180280; doi:10.1371/journal.pone.0107232)
Supplement: Table S5 — Predicted transcriptional regulators encoded by the Lactobacillus rossiae DSM 15814T genome. (DOCX) [file pone.0107232.s011.docx]

**Table S5.** Predicted transcriptional regulators encoded by the *Lactobacillus rossiae* DSM 15814^T^ genome.

| **ORF** | **Gene** | **Family** | **ORF** | **Gene** | **Family** |
| --- | --- | --- | --- | --- | --- |
| LROS_0007 | Predicted N-ribosylNicotinamide CRP-like regulator | CRP-like | LROS_0436 | Transcriptional regulator | Crp/Fnr |
| LROS_0020 | Transcriptional regulator | MerR | LROS_0442 | Putative regulator of the mannose operon ManO |  |
| LROS_0022 | Transcriptional regulator | ArsR | LROS_0447 | Two component system response regulator CiaR |  |
| LROS_0033 | Putative transcriptional regulator | MerR | LROS_0455 | Transcriptional regulator | Rrf2 |
| LROS_0034 | Transcriptional regulator | Rrf2 | LROS_0467 | Transcriptional regulator CtsR | OmpR |
| LROS_0041 | L-rhamnose operon transcriptional activator | AraC | LROS_0469 | Transcriptional regulator | TetR |
| LROS_0051 | Regulatory protein GntR HTH | GntR | LROS_0473 | Transcriptional regulator | ArsR |
| LROS_0055 | Citrate lyase transcriptional regulator CitI |  | LROS_0564 | Transcription regulator [contains diacylglycerol kinase catalytic domain] |  |
| LROS_0060 | Malolactic regulator |  | LROS_0589 | Transcriptional regulator | MarR |
| LROS_0061 | Transcriptional regulator |  | LROS_0741 | (-) Regulator of proteolysis |  |
| LROS_0073 | Transcriptional regulator | MerR | LROS_0742 | Ribose operon repressor |  |
| LROS_0086 | Putative transcriptional regulator |  | LROS_0744 | Redox-sensitive transcriptional activator OxyR |  |
| LROS_0093 | DNA-binding response regulator | OmpR | LROS_0788 | Two component transcriptional regulator | AraC |
| LROS_0097 | Transcription regulator |  | LROS_0816 | Ethanolamine two-component response regulator |  |
| LROS_0105 | Transcriptional regulator | GntR | LROS_0832 | Transcriptional regulator ArcR essential for anaerobic expression of the ADI pathway | Crp/Fnr |
| LROS_0113 | Transcriptional regulator | XRE | LROS_0837 | Sugar-binding transcriptional regulator | LacI |
| LROS_0116 | Transcription regulator of multidrug efflux pump operon TetR | AcrR | LROS_0853 | DNA-binding response regulator | OmpR |
| LROS_0131 | Transcriptional regulator | RpiR | LROS_0864 | Transcriptional regulator |  |
| **ORF** | **Gene** | **Family** | **ORF** | **Gene** | **Family** |
| LROS_0132 | Putative transcriptional antiterminator | BglG | LROS_0865 | Putative transcriptional regulator |  |
| LROS_0137 | Ribose operon repressor | LacI | LROS_0914 | Transcriptional regulator | PadR |
| LROS_0146 | Transcriptional regulator | IclR | LROS_0915 | Transcriptional regulator | XRE |
| LROS_0147 | Sugar diacid utilization regulator SdaR |  | LROS_0944 | Transcriptional regulator | LysR |
| LROS_0192 | Transcriptional regulator | TetR | LROS_0950 | Transcriptional regulator putative |  |
| LROS_0223 | Response regulator |  | LROS_0953 | Transcriptional modulator of MazE/toxin MazF |  |
| LROS_0230 | Regulatory protein | DeoR | LROS_0964 | Transcription regulator putative |  |
| LROS_0241 | Transcriptional regulator | TetR | LROS_0970 | HTH transcriptional regulator | TetR |
| LROS_0246 | Two-component response regulator SA14-24 | OmpR | LROS_0975 | HTH transcriptional regulator | TetR |
| LROS_0247 | Two-component sensor kinase | OmpR | LROS_0977 | Tetracycline repressor protein |  |
| LROS_0251 | Transcriptional regulator |  | LROS_0982 | Transcription regulator putative |  |
| LROS_0256 | Transcriptional regulator | HxlR | LROS_1000 | Positive transcriptional regulator | MutR |
| LROS_0261 | Transcriptional regulator | HxlR | LROS_1001 | Positive transcriptional regulator | MutR |
| LROS_0265 | Transcriptional regulator | TetR | LROS_1002 | Positive transcriptional regulator | MutR |
| LROS_0270 | Transcriptional regulator | TetR | LROS_1003 | Positive transcriptional regulator | MutR |
| LROS_0276 | Transcriptional regulator | MerR | LROS_1018 | Transcriptional regulator | MarR |
| LROS_0283 | Transcriptional regulator | ArsR | LROS_1034 | Transcriptional regulator | MerR |
| LROS_0287 | Regulatory protein | MerR | LROS_1042 | Transcriptional attenuator LytR-CpsA-Psr | F2 |
| LROS_0293 | Phosphate regulon transcriptional regulatory protein PhoB (SphR) | OmpR |  |  |  |
|  |  |  | LROS_1069 | Transcriptional regulator | LacI |
| LROS_0306 | Regulatory protein | TetR | LROS_1092 | Transcriptional regulator | TetR |
| LROS_0393 | Transcriptional regulator | XRE | LROS_1095 | Transcriptional regulator | TetR |
| **ORF** | **Gene** | **Family** | **ORF** | **Gene** | **Family** |
| LROS_0420 | Predicted transcriptional regulator of N-Acetylglucosamine utilization | GntR | LROS_1096 | Transcriptional regulator |  |
| LROS_0434 | Cell envelope-associated transcriptional attenuator LytR-CpsA-Psr | F2 | LROS_1113 | Transcriptional regulator/sugar kinase xylose operon regulator |  |
|  |  |  | LROS_1123 | AraC-like transcriptionalregulator |  |
|  |  |  | LROS_1125 | Predicted transcriptional regulator |  |
| LROS_1137 | Transcription regulator |  | LROS_1640 | Predicted transcriptional regulator |  |
| LROS_1161 | Transcriptional regulator | TetR | LROS_1644 | Transcriptional regulator |  |
| LROS_1186 | Transcriptional regulator | MerR | LROS_1648 | Transcriptional regulator | PadR |
| LROS_1190 | DNA-binding response regulator | AraC | LROS_1651 | Transcription regulator YobV *B. subtilis* |  |
| LROS_1248 | Transcriptional repressor of arabinoside utilization operon | GntR | LROS_1653 | Transcriptional regulator | XRE |
| LROS_1250 | Transcriptional regulator ArsR |  | LROS_1657 | Transcriptional regulator | TetR |
| LROS_1254 | Two-component response regulator |  | LROS_1659 | Transcriptional regulator |  |
| LROS_1257 | Transcriptional regulator | AraC | LROS_1666 | Transcriptional regulator | PadR |
| LROS_1276 | Malolactic regulator |  | LROS_1671 | Transcriptional regulator |  |
| LROS_1280 | Transcriptional regulator | TetR | LROS_1675 | Transcriptional regulator | PadR |
| LROS_1281 | Putative cell-cycle regulation histidine triad protein |  | LROS_1685 | Multidomain transcriptional regulator |  |
| LROS_1347 | Transcriptional regulator | GntR | LROS_1712 | Catabolite control protein A (CcpA) | LacI/GalR |
| LROS_1368 | Maltose operon transcriptional repressor MalR | LacI | LROS_1713 | Negative transcriptional regulator-copper transport operon |  |
| LROS_1379 | Transcriptional regulator | XRE | LROS_1722 | Transcriptional regulator | AcrR |
| LROS_1403 | FNR-like transcriptional regulator |  | LROS_1765 | Transcriptional regulator | MarR |
| LROS_1411 | Regulatory protein | LysR | LROS_1778 | Transcriptional regulator | PadR |
| **ORF** | **Gene** | **Family** | **ORF** | **Gene** | **Family** |
| LROS_1414 | Transcriptional regulator group III | Rrf2 | LROS_1781 | Stress-responsive transcriptional regulator PspC |  |
| LROS_1415 | Transcriptional regulator |  |  |  |  |
| LROS_1421 | Transcriptional regulator | TetR | LROS_1817 | Arginine pathway regulatory protein ArgR repressor of arg regulon |  |
| LROS_1430 | Transcriptional regulator | TetR | LROS_1881 | Transcriptional regulator | MerR |
| LROS_1431 | Transcriptional regulator | DeoR | LROS_1884 | Transcription regulator |  |
| LROS_1438 | Xylose operon regulator |  | LROS_1907 | Response regulator |  |
| LROS_1443 | Putative transcriptional regulator | MarR | LROS_1913 | Transcriptional regulator | HxlR |
| LROS_1467 | Negative transcriptional regulator-copper transport operon |  | LROS_1935 | TetR family regulatory protein of MDR cluster | TetR |
| LROS_1471 | Mn-dependent transcriptional regulator MntR |  | LROS_2026 | Transcription regulator |  |
| LROS_1475 | Putative regulatory protein |  | LROS_2039 | Transcription regulator |  |
| LROS_1486 | Transcriptional regulator | XRE | LROS_2040 | Predicted transcriptional regulator |  |
| LROS_1492 | Transcriptional regulator | Cro/CI | LROS_2045 | DNA-binding response regulator | AraC |
| LROS_1493 | Transcriptional regulator | MarR | LROS_2048 | DNA-binding response regulator | LuxR |
| LROS_1511 | Transcriptional regulator | GntR | LROS_2066 | Transcriptional regulators | LysR |
| LROS_1512 | Transcriptional regulator |  | LROS_2157 | Heat-inducible transcription repressor HrcA |  |
| LROS_1515 | Response regulator |  | LROS_2229 | Transcriptional regulator | MarR |
| LROS_1539 | Regulatory protein | RecX | LROS_2234 | Transcription regulator |  |
| LROS_1562 | Hcp transcriptional regulator HcpR | Crp/Fnr | LROS_2239 | Response regulator | LytR/AlgR |
| LROS_1586 | Transcriptional regulator |  | LROS_2249 | Signal transduction histidine kinase regulating citrate/malate metabolism | CitB |

| **ORF** | **Gene** | **Family** | **ORF** | **Gene** | **Family** |
| --- | --- | --- | --- | --- | --- |
| LROS_1588 | Transcriptional regulator | HxlR | LROS_2266 | Regulatory protein LacI: Periplasmic binding protein/LacI transcriptional regulator | LacI |
| LROS_1600 | Response regulator | LytR/AlgR | LROS_2298 | Regulatory protein | MerR |
| LROS_1601 | Transcriptional regulator | LacI | LROS_2302 | Predicted transcriptional regulator |  |
| LROS_1606 | Transcriptional regulator |  | LROS_2327 | TPR domain transcriptional regulator | Cro/CI |
| LROS_1621 | Organic hydroperoxide resistance transcriptional regulator |  | LROS_2329 | Transcriptional regulator | XRE |
| LROS_1623 | Transcriptional regulator | XRE | LROS_2377 | Alkaline phosphatase synthesis transcriptional regulatory protein PhoP | OmpR |
| LROS_2404 | Transcription regulator (putative) |  | LROS_2673 | Transcriptional regulator | HxlR |
| LROS_2417 | Peroxide stress regulator PerR | FUR | LROS_2731 | PurR: transcription regulator associated with purine metabolism | PurR |
| LROS_2439 | Galactose operon repressor | GalR-LacI | LROS_2752 | Predicted transcriptional regulator |  |
| LROS_2445 | Transcriptional regulator | TetR | LROS_2755 | Transcriptional regulator | TetR |
| LROS_2474 | Transcriptional regulator | MarR | LROS_2783 | Transcriptional regulator |  |
| LROS_2648 | Transcriptional regulator | ArsR | LROS_2785 | Transcriptional regulator |  |
| LROS_2651 | Transcriptional regulator | MarR | LROS_2800 | Transcriptional regulator |  |
| LROS_2658 | Transcription regulator |  | LROS_2808 | Transcriptional regulator | XRE |
| LROS_2671 | Transcriptional regulator | TetR | LROS_2828 | Transcriptional regulator | TrmB |
