# Supplementary material for: Lactobacillus rossiae, a Vitamin B12 Producer, Represents a Metabolically Versatile Species within the Genus Lactobacillus
Source: PLoS One. 2014 Sep 29;9(9):e107232. doi: 10.1371/journal.pone.0107232 (PMC4180280; doi:10.1371/journal.pone.0107232)
Supplement: Text S1 — Results - Supporting Information. (DOCX) [file pone.0107232.s012.docx]

**Results - Supporting Information**

**Amino acid biosynthesis, catabolism and proteolytic system**

Regarding the metabolism of free amino acids, the *L. rossiae* genome is predicted to specify a complete arginine deaminase (ADI) pathway (Table S3), of which homologues have been observed only in several *Lactobacillus* species (e.g. *L. amylovorus*, *L. acidophilus*, *L. fermentum*, *L. sakei* subsp. *sakei*, *L. brevis*, *L. buchneri*, *L. kefiranofaciens*) (Supporting Figure S4). This pathway consists of three enzymes: arginine deiminase (ADI, EC 3.5.3.6, LROS_0827), catabolic ornithine transcarbamoylase (cOTC, EC 2.1.3.3, LROS_0828) and carbamate kinase (CK, EC 2.7.2.2, LROS_0831) and a membrane transport protein (LROS_0829), which catalyses the electro neutral exchange between arginine and ornithine (Figure S4).

In depth analysis of the proteolytic system of dairy lactic acid bacteria (e.g., *Lc. lactis* and *L. helveticus*) showed that enzymes responsible for the liberation of free amino acids are grouped into three categories: (i) extracellular or cell-wall associated proteinases that hydrolysed environmental proteins into oligopeptides; (ii) specific transporters to take up oligo-, di- and tri-peptides and free amino acids from the environment; and (iii) intracellular oligopeptidases and peptidases, which cleave oligopeptides and peptides into amino acids. With respect to oligopeptide transport, *L. rossiae* is predicted to encode two complete transport systems, Opp (each consisting of the gene products of five genes, *oppD, oppF, oppB, oppC* and *oppA*), organized in two separate gene clusters (LROS_1741- LROS_1745 and LROS_1011-1015). Also, ABC transporters predicted to be specific for uptake of methionine (LROS_0717-0719), glutamine (LROS_2199), and L-proline, glycine and betaine (LROS_2757-2758) were present. In addition, genes encoding a putative di/tripeptide permease (LROS_0413), lysine permease (LROS_0457, LROS_0834), glutamate transport (LROS_1734-1737); D-serine/D-alanine/glycine transporters (LROS_1299, LROS_1804), branched-chain amino acid transporters (LROS_1442; LROS_1672; LROS_2676), cysteine transport system permease (LROS_1590), arginine ornithine/antiporter (arcD, LROS_0829); glutamate/gamma-aminobutyrate antiporter (LROS_0729) and generic amino acid permeases (e.g., LROS_0517; LROS_0523; LROS_1030; LROS_1105; LROS_1729; LROS_1995; LROS_2395-96; LROS_2822) were also identified.
