# Supplementary material for: Lactobacillus rossiae, a Vitamin B12 Producer, Represents a Metabolically Versatile Species within the Genus Lactobacillus
Source: PLoS One. 2014 Sep 29;9(9):e107232. doi: 10.1371/journal.pone.0107232 (PMC4180280; doi:10.1371/journal.pone.0107232)
Supplement: Text S2 — Experimental Procedures - Supporting Information. (DOCX) [file pone.0107232.s013.docx]

**Experimental Procedures-Supporting Information**

**Biosynthesis of riboflavin, folate, thiamine and cobalamin**

Growth of *L. rossiae* DSM 15814^T^ was assayed in modified semi-defined medium (LDMIIIG) (pH 5.6) [1], which was supplemented with 0.1 % Tween 80 and 1 % peptone. The modification was the use of mineral salts at one tenth of the concentration of the original medium. To study the effect of culture conditions on vitamin biosynthesis, *L. rossiae* DSM 15814^T^ was grown in LDMIIIG without riboflavin, folate, thiamine, or cobalamin. Twenty-four hours-old cells of *L. rossiae* DSM 15814^T^ grown in SDB medium [2] were inoculated (4 % v/v) in a riboflavin-, folate-, thiamine-, or cobalamin- free LDMIIIG medium, and transferred three times in the same medium. In order to analyze the production of cobalamin, DSM 15814^T^ was grown in anaerobic conditions and the medium was supplemented with 0.5 % of glycerol [3]. Growth was allowed for 18 h at 30°C. Growth was determined by measuring the optical density at 620 nm (OD_620_) and pH of the growth medium at various times. Each fermentation was carried out in triplicate.

**Cobalamin detection and quantitative bioassay**

To demonstrate cobalamin production by *L. rossiae* DSM 15814^T^, a bioassay was performed using *Lactobacillus leichmannii* ATCC 7830 (formerly *Lactobacillus delbrueckii* subsp. *lactis* ATCC 7830), a strain that requires B_12_ for growth [4]. Cell extract (CE) of *L. rossiae* DSM 15814^T^ and *L. plantarum* DC400 were prepared according to the protocol of Taranto *et al*. [5]. Different media were used to evaluate the cobalamin content in *L. rossiae* CE: vitamin B12 Assay Medium (B12-free) (Fluka, Sigma-Aldrich Chemie, St. Louis, MO); B12-free with increasing concentration (0, 0.025, 0.05, 0.075, 0.1, 0.125, 0.15 ng) of vitamin B12 standard (Fluka); and B12-free added with CE from *L. rossiae* or *L. plantarum* DC400. Cells of *L. leichmannii* ATCC 7830 grown on MRS broth (Oxoid Ltd.) at 37°C for 24 h were harvested by centrifugation, washed twice in sterile phosphate buffer 50 mM pH 7.0, centrifuged, re-suspended at the cell density of ca. 8.0 log colony forming units (CFU)/ml and used to inoculate the different media. The growth was determined by measuring the optical density at 620 nm (OD_620_) and the values of pH after 24h of growth at 37°C. Each fermentation was carried out in triplicate.

**Protease and peptidase activities**

Proteinase activity was measured using wheat albumins and globulins as substrates. The level of protein hydrolysis was estimated by Reversed-phase high-performance liquid chromatography (RP-HPLC) and amino acid analyses [6]. General aminopeptidase type N (EC 3.4.11.11; PepN), proline iminopeptidase (EC 3.4.11.9; PepI), X-prolyl dipeptidyl aminopeptidase (EC 3.4.14.5; PepX), endopeptidase (EC 3.4.23; PepO), and prolyl endopeptidyl peptidase (EC 3.4.21.26; PEP) activities were determined by the use of Leu-p-nitroanilides (p-NA), Pro-p-NA, Gly-Pro-p-NA, and Gly-Pro-Ala, Z-Gly-Gly-Leu-p-NA, and Z-Gly-Pro-NH-trifluoromethylcoumarin (AMC) substrates, respectively. The assay mixture contained 900 μl of 2.0 mM substrate in 0.05 M potassium phosphate buffer, pH 7.0, and 100 μl of cytoplasmic extract. The mixture was incubated at 30°C for 1 h, and the absorbance was measured at 410 nm. The data were compared to standard curves set up by using p-nitroaniline. Peptidase activity on Z-Gly-Pro-AMC was determined by measuring the fluorescence at excitation and emission wavelengths of 400 and 505 nm, respectively. Tripeptidase (EC 3.4.11.4; PepT), dipeptidase (EC 3.4.13.11; PepV), prolidase (EC 3.4.13.9; PepQ), and prolinase (EC 3.4.13.8; PepR) activities were determined using Leu-Leu-Leu, Leu-Leu, Val-Pro, and Pro-Gly substrates, respectively [6].

**Arginine catabolism assay, cell survival and biosynthesis of GABA from glutamic acid**

*L. rossiae* DSM 15814^T^ was propagated for 24 h at 30 ^o^C in modified MRS broth (Oxoid, Basingstoke, Hampshire, England) with the addition of fresh yeast extract (5 %, vol/vol) and 28 mM maltose to a final pH of approximately 5.6. Before cells were used for the arginine catabolism assay, they were sub-cultured (30 °C for 24 h) three times in MAM broth (tryptone, 10 g; yeast extract, 5 g; arginine, 3 g; KH2PO4, 0.5 g; MgSO4, 0.2 g; MnSO4, 0.05 g; Tween 80, 1 ml; glucose, 5 g; and H2O2, 1,000 ml [pH 6.0]) [6] with (adapted cells) or without (negative control) 17 mM arginine added. After cultivation, cells were harvested by centrifugation, washed with sterile distilled water, and resuspended in sterile distilled water to an optical density at 620 nm (OD620) of ca. 2.5. This bacterial suspension was used to inoculate (4 %, vol/vol) MAM broth with or without arginine (17 mM), followed by static incubation at 30 ^o^C for 72 h. The acidification kinetics were determined on-line by a pH-meter for a solid food matrix (pH-meter 507, Crison, Italy). Cell numbers were determined by plating on SDB agar medium at 30 °C for 72 h. The three enzyme activities which comprise the ADI pathway was also assayed as previously described [7].

**Statistical analyses**

All data were obtained at least in three replicates. The percentages were arcsine-transformed for data analyses. Analysis of variance (ANOVA) was carried out on transformed data, followed by separation of means with Tukey’s HSD, using Statistica for Windows statistical software (version 7.0).

**References**

1. Jones SE, Versalovic J (2009) Probiotic *Lactobacillus reuteri* biofilms produce antimicrobial and anti–inflammatory factors. *BMC microbiol* 9: 35.

2. Kline L, Sugihara TF (1971) Microorganisms of the San Francisco sourdough bread process. Appl Environ Microbiol 21: 459-465.

3. Santos F, Spinler JK, Saulnier DM, Molenaar D, Teusink B, et al. (2011) Functional identification in *Lactobacillus reuteri* of a PocR–like transcription factor regulating glycerol utilization and vitamin B12 synthesis. Microb Cell Fact 10: 55.

4. Horwitz W (2000) Official methods of analysis of AOAC International, 17th ed. AOAC International, Gaithersburg, Md.

5. Taranto MP, Vera JL, Hugenholtz L, De Valdez GF, SesmaF (2003) *Lactobacillus reuteri* CRL1098 produces cobalamin. J Bacteriol 185: 5643-5647.

6. De Angelis M, Cassone A, Rizzello CG, Gagliardi F, Minervini F, et al. (2010) Mechanism of degradation of immunogenic gluten epitopes from *Triticum turgidum* L. var. durum by sourdough lactobacilli and fungal proteases. Appl Environm Microbiol 76: 508-518.

7. De Angelis M, Mariotti L, Rossi J, Servili M, Fox P, Rollan G, Gobbetti M (2002) Arginine catabolism by sourdough lactic acid bacteria: purification and characterization of the arginine deiminase pathway enzymes from *Lactobacillus sanfranciscensis* CB1. Appl Environm Microbiol 68: 6193–6201.
